# Barcoding a Quantified Food Web: Crypsis, Concepts, Ecology and Hypotheses

**DOI:** 10.1371/journal.pone.0014424

**Published:** 2011-07-06

**Authors:** M. Alex Smith, Eldon S. Eveleigh, Kevin S. McCann, Mark T. Merilo, Peter C. McCarthy, Kathleen I. Van Rooyen

**Affiliations:** 1 Biodiversity Institute of Ontario and Department of Integrative Biology, University of Guelph, Guelph, Ontario, Canada; 2 Natural Resources Canada, Canadian Forest Service, Atlantic Forestry Centre, Fredericton, New Brunswick, Canada; 3 Department of Integrative Biology, University of Guelph, Guelph, Ontario, Canada; 4 Population Ecology Group, Forestry and Environmental Management, University of New Brunswick, Fredericton, New Brunswick, Canada; Dalhousie University, Canada

## Abstract

The efficient and effective monitoring of individuals and populations is critically dependent on correct species identification. While this point may seem obvious, identifying the majority of the more than 100 natural enemies involved in the spruce budworm (*Choristoneura fumiferana* – SBW) food web remains a non-trivial endeavor. Insect parasitoids play a major role in the processes governing the population dynamics of SBW throughout eastern North America. However, these species are at the leading edge of the taxonomic impediment and integrating standardized identification capacity into existing field programs would provide clear benefits. We asked to what extent DNA barcoding the SBW food web would alter our understanding of the diversity and connectence of the food web and the frequency of generalists vs. specialists in different forest habitats. We DNA barcoded over 10% of the insects collected from the SBW food web in three New Brunswick forest plots from 1983 to 1993. For 30% of these specimens, we amplified at least one additional nuclear region. When the nodes of the food web were estimated based on barcode divergences (using molecular operational taxonomic units (MOTU) or phylogenetic diversity (PD) – the food web became much more diverse and connectence was reduced. We tested one measure of food web structure (the “bird feeder effect”) and found no difference compared to the morphologically based predictions. Many, but not all, of the presumably polyphagous parasitoids now appear to be morphologically-cryptic host-specialists. To our knowledge, this project is the first to barcode a food web in which interactions have already been well-documented and described in space, time and abundance. It is poised to be a system in which field-based methods permit the identification capacity required by forestry scientists. Food web barcoding provided an effective tool for the accurate identification of all species involved in the cascading effects of future budworm outbreaks. Integrating standardized barcodes within food webs may ultimately change the face of community ecology. This will be most poignantly felt in food webs that have not yet been quantified. Here, more accurate and precise connections will be within the grasp of any researcher for the first time.

## Introduction

***‘What's the use of their having names the Gnat said, ‘if they won't answer to them?’***

***‘No use to them,’ said Alice; ‘but it's useful to the people who name them, I suppose. If not, why do things have names at all?’***

***‘I can't say,’ the Gnat replied. ‘Further on, in the wood down there, they've got no names.***

***Lewis Carroll, Through the Looking Glass***

The spruce budworm (*Choristoneura fumiferana*, SBW) is the most economically important insect species in eastern North America. Every 30–40 years, the species undergoes population outbreaks [1] that can result in damage to over tens of thousands of hectares, affecting hundreds of communities and costing many millions of dollars. For example, consider an outbreak of the scale of the one that peaked in the mid-1970’s – where SBW defoliation peaked at approximately 57 million ha [2]. If the impending outbreak due to affect eastern North America within the next few years reaches this magnitude, it would cost billions of dollars (A value approximated by dividing the average value of forest land ($/ha) from the contribution of forests to GDP by the total forested area in Canada in 2009 multiplied by 57 million ha [3]). Despite being a disruptive ecological force on a continental scale with impacts comparable to forest fires, there is currently no consolidated plan for managing budworm outbreaks [4]. Clearly, there are gross economic factors that ought to reinforce how important an understanding of the food web of organisms that depend on, and interact with, the spruce budworm is to our environment and our economy.

The population dynamics of the budworm can be mediated by insect parasitoids, (wasps (Braconidae [5], Ichneumonidae [6], Chalcidoidea [7]) and flies (Tachinidae [8])) operating at two trophic levels (primary parasitoid and secondary (or hyper-) parasitoid). This array of just over 100 primary and secondary parasitoids that prey upon the budworm, and competing Lepidoptera [5] can decrease the magnitude of budworm outbreaks [9] and are therefore critical components of this complex food web. From a community ecological perspective, the parasitoids within the SBW food web can canalize energy and nutrient flow dependent on whether they attack many hosts (a generalist) or a small number of (or single) host species (a specialist) in the ecosystem. Currently, the majority of the parasitoids in the SBW food web are considered to be generalists [9].

A thorough understanding of the SBW food web is then contingent on the efficient and accurate identification of the individual species within that food web (who is who, and who eats whom). Currently, this identification involves highly specialized taxonomic expertise [5], [6], [7], [8], [10] and expensive long-term rearing programs [9]. This diversity- enforced bottleneck is not unique to this system but is a global phenomenon. Consider the magnitude of the insect diversity problem. Nearly ¼ of all animal species are insects; we expect that up to ¼ of all insects are parasitoids and, furthermore, it is within this enormous block of life that we are most exposed to the taxonomic impediment [11]. While only 10% of all insect species are described [12] – identified parasitoid diversity may be as low as 1% [13], [14]. Thus, there is a grave need for accelerated identifications within these diverse and economically important groups.

The majority of the parasitoids in the SBW food web are currently considered to be generalists [9]. However during comparable studies of parasitoid diversity in tropical food webs, the iterative process of barcoding a parasitoid fauna associated with rearing records and permanent collections increased the estimates of host-specialization and drastically reduced the frequency of the generalist strategy [15], [16], [17]. Specifically, the majority of the morphologically cryptic, presumably polyphagous species dissolved into monophagous species groups supported by ecology, and both mitochondrial and nuclear genetic divergences.

In this study, we were interested in asking if applying the iterative [15] process of DNA barcoding to the parasitoid individuals within the temperate SBW food web would produce the same fraction of newly-revealed cryptic species as in tropical food webs. In the Area de Conservacion de Guanacaste in Costa Rica, approximately 25% of the ‘named’ species involved in parasitizing larval Lepidoptera were in fact genetically divergent – and are now considered to be different species [18]. We predicted that some proportion of the parasitoid fauna in the SBW food web would also be revealed as morphologically cryptic, but genetically distinct – but that this rate would be less than was uncovered in the tropics.

If this prediction was supported, how would such an increase in diversity affect food web structure? The SBW is an exceptional dataset in food web ecology in that it has been collected in multiple locations through time, has been accessioned in a fashion that is amenable to the recovery of multiple fragments of DNA (both nuclear and mitochondrial), and it has been measured in space and time for not just diversity, but for species abundances as well [9]. Thus, by inserting a barcoding component into the quantified SBW food web we are in the exceptional position of asking whether identifying units using barcoding affects the structure of a food web that varies in space, time and abundance.

Historically, food web ecology has suffered from a misunderstanding of rules and principles because of problems with resolution – not just “who eats whom” but “who is who” – in node selection [19], [20]. To properly understand connectivity and energy flow in ecological communities, a pragmatic and repeatable resolution of taxa or nodes is required.

Molecular markers have had a long history of being used to identify ‘who is who’, and have also recently been used in several instances to help identify ‘who eats whom’ [21]. For instance, Garros et al [22], used DNA barcodes to identify blood meals of malarial mosquitoes. Clare et al [23], used DNA barcodes generated from fragments retrieved from bat guano to help construct the diet of a generalist top predator. Gariepy et al [24] used multiplex PCR to estimate levels of parasitism and parasitoid species composition. Hardy et al [25] used 16S DNA sequences and microarrays to delineate carbon flow in an Australian riverine system. Corse et al [26], used group specific primers to amplify DNA from the diet of freshwater cyprinid species. Locke et al [27] used DNA barcodes and nuclear sequences to identify cryptic host and tissue specialization of Diplostomoidea (Platyhelminthes: Digenea) parasitizing freshwater fishes in Canada. Kaartinen et al [28] were the first to use CO1 DNA barcodes and ITS2 sequences to test species memberships and connections in a food web derived from leaf-mining Lepidoptera and gall-inducing Hymenoptera occurring on *Quercus robor* in northern Europe.

Our molecular ecological characterization of a food web is unique in that it involves a very diverse system, based on outbreaking host species of enormous economic importance, where all species are from a food web that has already been characterized in abundance, space and time [9], and that the molecular comparisons made include the standardized DNA barcode region – thereby permitting direct comparison to other systems. We are therefore able to compare barcode-based analyses of food web structure to previous analyses where nodes (species) were identified using principally morphological methods. Specifically, we were able to ask whether the ‘bird feeder effect’ (that fluctuations in budworm density will cause diversity cascades such that more higher order parasitoids will occur at higher SBW densities [9]) is amplified, reduced or not affected when the units of higher order diversity are enumerated using DNA barcodes, nuclear genes, host records and morphology rather than morphological identifications alone.

The erection of a species hypothesis within a morphologically cryptic taxon based on DNA barcodes ought to be supported by additional, independent nuclear marker(s) [16], [17], [29], [30]. Even with small sample sizes, uncovering a matching split between two independent loci by chance is low [31]. We used several rDNA loci (ITS1, ITS2 and 28S-D2). Some have suggested that the presence of compensatory base pair changes (CBCs) in the secondary structure of the ITS2 region can be used as a proxy to identify sexually incompatible pairs [32], [33]. CBCs occur when both nucleotides of a paired site mutate but the pairing remains stable. If CBCs do correlate with (or cause) sexual incompatibility – they could be a molecular, ‘holy grail’ [34] for identifying species as defined by the biological species concept [35]. We tested whether the units of food web diversity were differentiated differently using morphology, barcodes and ITS2 CBCs for a subset of the food web diversity.

Finally, we considered the importance and ramifications of species concepts on food web node identification whether determined using morphology, genetic information or ecology in either an integrative or a separate fashion. We make specific recommendations regarding the erection of ‘species hypotheses’, the importance of recognizing both Type I and II errors in formulating and testing these hypotheses, and the likelihood that barcodes will solve the species problem.

We found that barcoding our quantified insect food web resulted in an approximate 41% increase in the number of nodes within the web and thus the connectence of the web was reduced. However, previous conclusions regarding the basic structure of the web in different forest plots were not significantly altered by identifying nodes via molecular tools rather than by strictly morphological ones.

## Results

From 1983 to 1993 there were 12, 292 parasitoid specimens, from 98 species collected at three collection sites in New Brunswick, Canada. From this dataset, 1,710 specimens were sampled for barcoding between 2007 and 2009. Of these, CO1 fragments were generated for 1,492 specimens (12.1% of the total collected and 87.3% of the specimens extracted), 28S for 573 specimens (38.4% barcoded), ITS2 for 80 specimens (5.4% barcoded) and ITS2 for 93 specimens (6.2% barcoded).

In one case (*Mesopolobus verditer* - EE-13510-86 P1) we amplified the CO1 of a bacterial endosymbiont rather than of the insect host.

Specimens from 11 genera within five families of parasitoid wasps displayed a characteristic 6 bp deletion in CO1 in the 155^th^ and 156^th^ amino acids of the barcoding region (Chalcididae (*Conura*), Encyrtidae (*Copidosoma*), Eulophidae (*Aprostocetus*, *Baryscapus*, *Elachertus*, *Elasmus, Euplectrus* and *Pediobius*), Perilampidae (*Perilampus*) and Pteromalidae (*Mesopolobus*, *Pteromalus*)). This six base pair deletion occurs within the third internal loop, (likely at the meeting with the fifth membrane-spanning helix), is in frame, has no anomalous amino acid variation following the deletion and occurs within all GenBank sequences from this family. Agarose gels made of the CO1 amplification contained no anomalous secondary bands, and there was no evident systematic heteroplasmy within the trace files. Thus we consider that these gene fragments represent true mitochondrial products and not pseudogenes.

Specimens from the genus *Copidosoma* (Encyrtidae; Encyrtinae) were also characterized by a 1 base pair deletion that, if unrecognized, would place the alignment out of frame and result in stop codons, and is likely a pseudogene or NUMT [36]. Interestingly, specimens from each provisional species displayed this deletion. While there were no corresponding divergences within the 28S, there were three ITS2 groups. Further work is clearly required on this species and for the purposes of this food web analysis, we considered this species to be two. We used the putative pseudogene as the CO1 markers for this species in these analyses.

Barcoding the reared specimens revealed 32 individual insect specimens (2.1% of barcoded total) which had apparently been misidentified morphologically (Figure S1).

**Figure 1:**
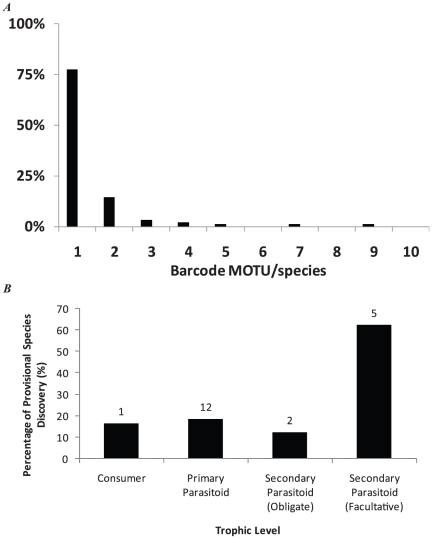
Proportions of cryptic diversity revealed through barcoding the SBW quantified food web. A) Proportion of barcoding MOTU (provisional species) uncovered within each morphologically described species. B) Proportional representation of the identification of cryptic diversity within each level of the SBW food web. The number above each bar represents the absolute number of cases of cryptic diversity within each category.

**Figure 2:**
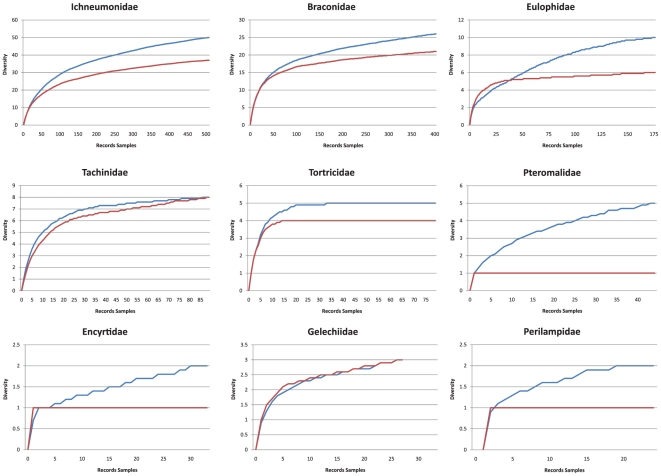
Diversity accumulation curves of specimens measured using traditional morphology (species) or using single CO1 variation (barcodes). Accumulation curves calculated using BOLD [74] following 20 randomizations. Blue lines represent barcode diversity, red lines represent morphologically named taxonomic diversity.

**Figure 3:**
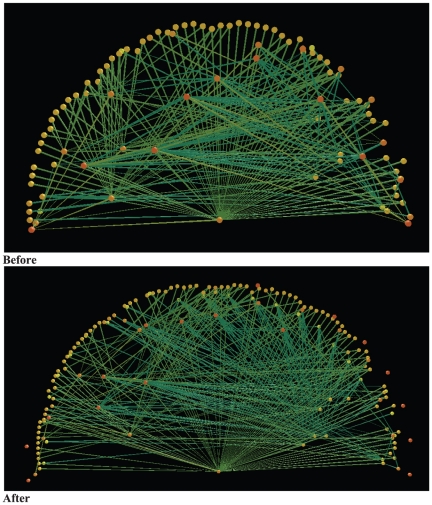
Food web representation where nodes are described morphologically as in [**9**] (BEFORE), and where nodes were delineated using barcodes (AFTER). Diversity has increased (Nodes (N) increased from 110 to 156 (a 41% increase)) and links (L) from 336 – to 449) and the connectence (Connectance (L/N^2^)) has been reduced (from 0.03 to 0.02). Nodes are unlabelled, and the SBW is the center of the fan. Image produced with FoodWeb3D, written by R.J. Williams and provided by the Pacific Ecoinformatics and Computational Ecology Lab (www.foodwebs.org, [73]).

The majority of the species barcoded produced CO1 barcodes that displayed little or no intra-specific variation (Figure 1). There were 22 cases where a named species (or morphospecies; out of the 91 named species barcoded (Table S1), 24%) was revealed to contain deep mitochondrial divergences that were suggestive of multiple species existing within the currently acknowledged name (Figure 2, Figure S1). These cryptic provisional species were distributed amongst all trophic levels (Figure 1) and decreased the connectence of the food web (Figure 3). Specifically, delineating species via barcodes caused an increase in diversity (Nodes (N) increased from 110 to 156 (an increase of 41% from nodes calculated based on morphology as in [9]) and links (L) from 336 – to 449) while the connectence (Measured as L/N^2^) was reduced from 0.03 to 0.02.

Barcoding resulted in the re-evaluation of 10 polyphagous generalist species into 30 provisional species including more monophagous host-specialists (Table S2). While sample sizes are small for some of these comparisons, 16 (53.3%) of these new species were host-specialists while 14 (46.7%) remain generalists.

From the newly recognized provisional species, (possibly representing 64 cryptic species) we selected cases to amplify a ribosomal DNA marker (ITS2 (n = 36 of the provisional species) or 28S-D2 (n = 40 of the provisional species) to test for congruence of the divergence within the species named. (GenBank accessions for all sequences analyzed here are included in Table S1).For those species where we generated ITS2 data (n = 39), we found that 87.2% of the CO1 groups tested also displayed ITS2 variation. In 5 cases there was no ITS2 variation in corroborate to CO1variation, and in one case, there was evident ITS2 variation within a CO1 invariant set. In addition to these ITS2 sequence based comparisons, we also made pairwise comparisons for CBCs. In only two cases (5.1% of those species examined with ITS2), were provisional species identified by sequence variation in CO1 and ITS2 – supported by the presence of CBCs (Table S3).

For those species where we generated 28S data (n = 45), 88.9% of the CO1 groups we tested also displayed D2 variation. In 5 cases there was no D2 variation that corroborated the CO1variation (Table S3). GenBank accessions for D2 sequences analyzed here: HQ025168-HQ025800. See Table S3 for a comparison of provisional species splits across CO1 and the rDNA loci.

**Figure 4:**
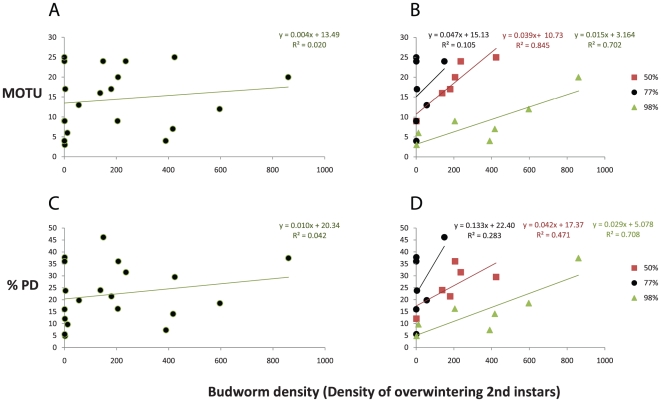
Food-web diversity measured using DNA barcodes and budworm density. Here diversity was calculated as MOTU (A&B), or PD (C&D) and separate trends were plotted for the nearly homogenous (98% balsam fir), intermediate (77% balsam fir) and the most heterogeneous site (50% balsam fir) (C&D). The ‘bird feeder” effect predicts that the diversity of parasitoids will increase with the budworm density. As was found in [9], the slopes of these lines (in the 50% and 98% balsam fir plots) are significantly different from the null hypothesis of 0 – supporting the bird feeder effect. As in [9], the data were de-trended to remove potential temporal autocorrelations but as this yielded consistent results, the original comparisons are illustrated here.

One of the principle predictions of the “bird feeder effect” (that parasitoid diversity should track SBW abundance) was tested when parasitoid diversity was determined using barcode provisional species (molecular operational taxonomic units (MOTU) [37]) or the phylogenetic diversity (PD) [38] contained within a barcode neighbor-joining tree. We found that measuring the diversity of the parasitoid community using either measure of barcode diversity did not affect the prediction of the model – namely diversity increased with increasing budworm density (Figure 4) and that these effects were more evident in the more heterogeneous environments.

## Discussion

### SBW Food Web Diversity

Barcoding the SBW food web resulted in an increased appreciation of diversity across all trophic levels (Figures 1, 2). Species presumed to be single biological units were revealed by examination of genetic divergences for both single mitochondrial and multiple nuclear loci to be multiple units. These deep genetic divergences were often, but not always, coincident with different host records. The proportion of cryptic diversity uncovered was greater in smaller taxa (Eulophidae, Pteromalidae), than in larger taxa (Tortricidae, Tachinidae, Ichneumonidae). The importance of body size to the proper characterization of a food web is well known [39], [40], [41], [42], and if smaller organisms are more likely to contain cryptic diversity – then the relative importance of body size may be even more challenging to quantify if it is particularly confounded by problems of species resolution and identification amongst the smallest size classes. For instance, theory predicts that small body size individuals ought to be lower in the food web [41]. In our web, the highest order parasitoids are often amongst the smallest individuals – often containing a large preponderance of cryptic diversity. If different aspects of food web structure are prone to an effect of body size then there are likely to be theoretical repercussions when web resolution is determined via such a standardized approach as DNA barcodes.

We found it very interesting how the rate of discovery of cryptic species here in the temperate Acadian forest is so similar (24% of presumed single species decayed into multiple provisional species following barcoding) to the rate of cryptic parasitoid species revealed in a tropical environment [25% –15]. Indeed, similar findings have been recently reported from northern Europe where 31% of species designations in leaf-mining Lepidoptera and gall-inducing Hymenoptera occurring in the *Quercus robor* food web changed following barcoding [28].The discovery of cryptic diversity, particularly in parasitoid insects, is not singularly a story from the diverse tropics. It is a real, if unappreciated phenomena, in temperate [43], and even northern [29], systems as well.

**Figure 5:**
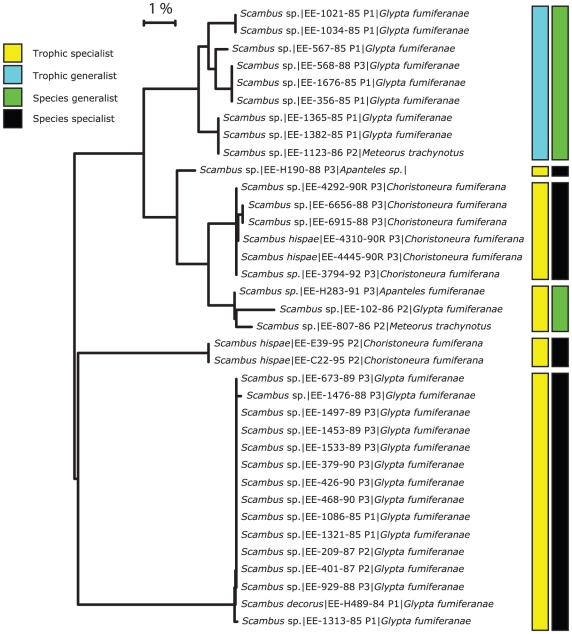
Neighbour-Joining tree of specimens from the parasitoid *Scambus.* (Ichneumonidae; Pimplinae) [i.e. a secondary parasitoid whose host records include both primary parasitoids and consumers] (determined morphologically). Tip labels morphological species|Sample ID|Host species|Barcode MOTU. Upon barcoding, the species split into 6 provisional species. Of these, 5 are trophic specialists, one appears to be a trophic specialist, while 4 are species specialists and two are species generalists. One of the provisional species appears to remain a trophic and species generalist.

Consider one example where a parasitoid was previously considered a species and trophic generalist (*Scambus hispae* (Ichneumonidae; Pimplinae) – but often only identified as *Scambus* sp.): a facultative secondary parasitoid (Figure 5). Upon barcoding, the species split into 6 provisional species. Of these, 5 are trophic specialists, one appears to be a trophic generalist, while 4 are species specialists and two are species generalists. One of the provisional species remained both a trophic and species generalist.

Our analysis permitted us to re-test a specific quantitative aspect that recent theory suggested was of critical importance to food web structure – the “bird-feeder effect”. In our estimation, one of the most intriguing results presented here is that this measure of ecosystem function was not significantly affected by the increased nodal resolution offered by molecular ecology.

When all three sites are pooled, the relationship between barcode-estimated diversity and SBW density is noisy and appears neither significant nor apparent. However, when sites are plotted separately, the barcode-estimated diversity was lowest in the homogeneous plot and higher in the more heterogeneous plot (Figure 4) and furthermore there is a positive relationship between SBW abundance and parasitoid diversity – just as predicted by the “bird-feeder effect”. Does environmental heterogeneity apparently muddy the relationship between abundance and diversity, or is this an effect of measuring diversity via the barcode? The noise in the relationship may be due to barcode estimates of diversity resulting in a more fine grained estimation of biodiversity (See accumulation curves in Figure 2). In turn, this accentuated the differences between the three collection localities that differed in heterogeneity. For instance, in demonstrating the “bird feeder effect” [as in Figure 2 from reference 9], our analysis of diversity calculated using barcodes (either in MOTU or PD) was not significant (Figure 4 A&C). However, when each locality was analyzed separately, a strong relationship was evident using both MOTU and PD (Figure 4 B&D). This more fine-grained estimation of biodiversity revealed a steeper relationship between diversity and ecosystem function (measured as the “bird feeder effect”) in localities with greater heterogeneity in an essential resource (% of the stand comprised of balsam fir). Another (and not necessarily competing) explanation is likely due to the fact that Plot 2 (the 77% balsam fir plot) was sampled when the SBW populations were already declining to low levels, causing a cloud of points to occur near the y-axis, thus nullifying the overall increasing trend when all plots were pooled. If Plot 2 had been sampled over the same range of SBW densities as the other plots, there is little doubt that the overall relationship of pooled sites would be positive.

Food web theory also predicts that the most heterogeneous sites should have the greater numbers of host-generalists than specialists [9]. In ten cases, a species previously thought to be a host generalist is now considered to be a (or to contain a) host specialist (Table S1). Within these ten species, there may be 30 cryptic species and of these, 20 are not found at the most homogenous site (Table S2). Again, this finding supports the theoretical prediction.

### Generalists and Specialists

Our study is no different from others involving perceived mono- or polyphagy in that our use of the terms “specialist” and “generalist” ought to be understood to be placed within the context of a specific space and time. Clearly any species identified in the field as a specialist — regardless of the empirical approach — may not be an absolute specialist if all individuals within a population were followed for multiple generations or across multiple populations. Nonetheless, given this general ecological problem, it is important to recognize that our approach is actually biased towards finding generalists in that we intentionally selected specimens for barcoding to represent as broad a variety of the hosts as possible. If we had reared specimens from only one host species we would have been biased towards discovering specialists. However we have measured the prevalence of parasitoids within the most abundant herbivores in this system – and in the context of this study we are confident that we are identifying the major functional parings. Thus, we use the term specialists as a functional term here – and thus when primary hosts are low it does not presuppose that this parasitoid will be unable to host switch. Indeed, faced with extinction and starvation – we might expect them to ‘eat ‘anything available. Therefore, within the context of our collection, we are confident that we have captured the predominant links. Indeed, by selecting our parasitoids from as broad a variety of hosts as possible – we may have biased our detection towards uncovering generalists rather than specialists.

### PD & MOTU

It is important to note that our aim in using the neighbor-joining trees constructed here with a single marker is not to construct the most resilient nor accurate deep phylogeny. Rather, it was to use the PD approach parameterized with the standardized DNA marker to produce an estimate of ‘diversity’ independent of, but not unrelated to, the species question. Phylogenetic diversity (PD) [44] was measured as the evolutionary branch length spanned by a given set of species. In a more complete phylogeny, PD is expected to have important consequences for community assembly if close relatives exhibit greater ecological similarity than distant relatives [45]. As used here, a phylogenetically ‘close’ terminal is not necessarily the end result of the phylogenetic analysis – rather – the intended end result is to make transparent comparisons of diversity in a manner that is standardized (the accepted animal barcoding locus) in a fashion that is independent of species decisions. Such an analysis could be based on representative sequences – or all sequenced specimens (as here). When the conclusions of barcode based analyses have been compared between MOTU and PD [29], [46], [47] significant differences were not found between the two analyses. This was again the case here – while there were qualitative differences between the two results – they were not significant. Our results add to a growing literature demonstrating the power of using PD based on a standardized gene region (even a single marker) [46], [47], [48], [49].

### CBCs and species

Recently, several papers presented evidence that strongly supported the hypothesis that the internal transcribed spacer region 2 (between 5.8S and the large subunit (LSU, 28S)) can be used as a species level identifier across broad taxonomic groups [50], [51]. Specifically, the presence of even one CBC in ITS2 predicts a total lack of successful interbreeding [32]. An ITS2 pipeline has been suggested, similar to the Barcode of Life Datasystem (BOLD), which would make species identification very simple via the comparison of CBCs. Compensatory base changes (e.g. CG–AT), maintain pairing at corresponding sites and therefore secondary structures. Barcoding will have the greatest impact on food web ecology when the lineages it identifies equate as closely to independently evolving lineages as possible. Ultimately testing these lineages will necessarily be contingent upon multiple loci; however the initial identification of these lineages using mtDNA is a critical and useful first step [52]. To examine the hypothesis that compensatory base changes (CBCs) specifically within the ITS2 rRNA secondary structure are an effective method for delineating cryptic species, we examined multiple comparisons within our insect food web.

The CBC hypothesis for species identification is, by nature, comparative, and thus it is critical that the sequences being compared have sufficient variation so that compensatory mutations can be identified, but not so much variation that alignment is compromised [53]. Achieving this balance is a non-trivial problem. Our data suggest that the pipeline of identification is not yet mature for insects (at least from the families examined here) and while it may be eventually, without a larger group of secondary structure models for insects, one must depend on provisional estimates of folding and structure – and thus, at best, provisional estimates of CBCs.

In addition, we uncovered CBCs in only a very small proportion of the provisionally identified (barcoding, host, rDNA sequence divergence) new species sequenced here. While CBCs undoubtedly exist between many sexually-isolated species – we did not find them here frequently enough to warrant their discussion as a “holy grail” for species identification. Our species are not the only ones where sequence data from ITS2 has been used to identify cryptic species – but where CBCs are absent. Van Veen et al [54] has been cited as a pioneering study in the use of molecular markers to resolve the identification of ‘difficult’ parasitoid species. In the four cryptic species of *Alloxysta victrix* that were examined – there are CBCs present between only one of 6 possible pairwise comparisons (Table S4).

### Species, barcodes and concepts

Species hypotheses should not be formulated in the absence of a species concept [55]. In this work, we use a species concept derived from the evolutionary species concept [56] and the general lineage concept of species [57], implemented using the phylogenetic species concept [58] where we seek to identify individual, independent-evolutionary lineages as species using operational criteria that include molecular (DNA sequence data), morphological and ecological information.

A species concept tests an individual’s membership based upon different criteria (of which a barcode could be one). This depends on the *a priori* erection of a hypothesis; either 1) an individual is a member of species A (as in [59]), or 2) an individual is not a member of species A. In each case, the null is different (Null 1  =  separate species; Null 2  =  same species). Historically, the stress has always been on the p value approach (significance) and considering Type 1 error (failure to reject a null hypothesis when it is true). Emphasis has also been predominantly on the second hypothesis – the null of ‘same species’ approach. If the null cannot be rejected, Scenario 1 will increase species number while Scenario 2 will decrease species number. This leads to an underestimation of diversity and furthermore, it fosters an under appreciation for magnitude of importance for the Type II error (of accepting the same when they are separate).

**Figure 6:**
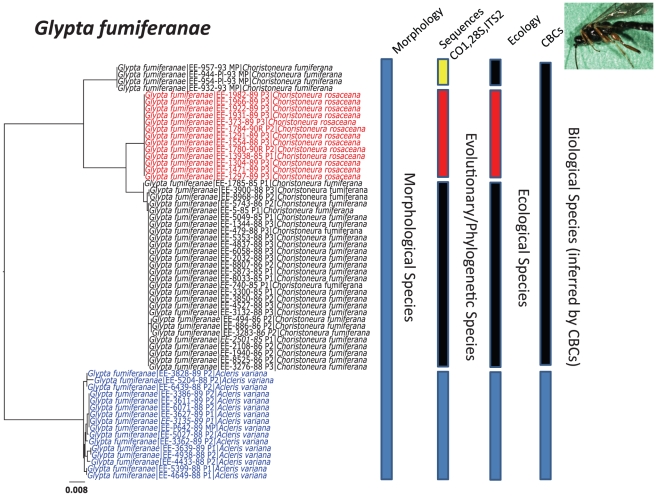
Species concepts and criteria as applied to one member of the SBW food web, *Glypta fumiferanae*. Evidence that this primary parasitoid (Ichneumonidae) is between 1 and 4 species is presented depending on which set of operational criteria one uses.

Consider the example of *Glypta fumiferanae* in Figure 6. Here, morphology suggests that all specimens belong to one species; the sequence data from three genes suggests that there are four species. Ecological differences of host species suggest there are three species. Finally, if we use the presence of complementary base pair changes (CBCs) to infer reproductive isolation (and the biological species concept) then the specimens currently named *G. fumiferanae*, are correctly considered to be two species. Prior to DNA evidence, there was no evidence to suggest different species (except for multiple hosts). Thus, H_o_ is that all individuals arise from one species, while H_1_ is that individuals from different hosts represent different species. In this scenario, the emphasis is on Type 1 error (incorrectly rejecting null when it’s true). However, this discussion to date has not identified the importance of sampling (how many specimens are sufficient to detect the pattern) and what to do if the sampling is fixed (i.e. in the scenario of an after-the-fact analysis using museum specimens – sample size is fixed by the number of specimens deposited and there are no more). How many individuals does it take to ensure that the species is well sampled? Whatever number this is (ideally more than 5 [60], 20 or more [61], or as high as several hundred [62]) in a retrospective analysis as described above we are unlikely to reach this number. Furthermore, one must also consider the “effect size” of the relationship being compared (nominally categorised as ‘small’ or ‘large’) – and what this may be in the context of confusing intraspecific morphological variation.

If sample sizes are small, statistical power is low – especially if the confidence level is maintained at 0.05 – we maintain that researchers should consider optimizing statistical thresholds (e.g., compromise analysis). We must explicitly consider the selection of null hypotheses as regards species testing. Specifically, within parasitoid communities, it may be beneficial to consider altering the default selection of the statistical null hypothesis. If sample sizes are low, but specimens are known to arise from different hosts, the null hypothesis ought to be that they are different species (if and until evidence is collected that unites them).

A DNA barcode is an epistemological tool, an evaluation criterion for identification and can act as a catalyst for discovery. It is not an ontological truth that defines a species. Is DNA barcoding a “solution” to the “species problem”; unequivocally no. Will it make future hypotheses regarding “who is who” (species membership) and then “who eats whom” more transparent and reproducible – unequivocally yes.

### Future

These analyses are a further contribution to a new approach to food web ecology. Food web ecology has always had to deal with the demons of resolution and how these affect the search for universal properties. When taxa are resolved at a very coarse scale or in a fashion that is not transparent and testable – the data underlying this search becomes extremely questionable [19]. Our approach to food web ecology is resolvable in a pragmatic and repeatable fashion and makes strides towards a very practical application of moving towards easily identified food web units.

In addition to extending the analyses presented here – we look forward to tightening now invisible connectences through the amplification of gene fragments from those parts of the collection that were not reared successfully (host fragments) – particularly in cases of hyperparasitoids where the intermediate host is unknown. Other approaches we will investigate in the future include the integration of molecular tools into new field collections where we will amplify the host species’ DNA from the gut-contents of the parasitoid – thereby both enabling the avoidance of the long times sometimes necessary for rearing completely to emergence – and potentially permitting one to map parasitoids to hosts even for those specimens arising from passive collections [63]. Furthermore, it will be interesting in the future to consider food web properties in addition to the number of nodes, links, and connectance [64].

The importance of developing barcode libraries for food webs crosses into other ecological disciplines and issues facing society. Increasing public awareness of the biodiversity crisis has placed a new set of demands upon resource managers. One obvious aspect of this looming issue is to delineate diversity. Barcoding techniques play a clear and obvious role here as they are allowing us to quantify diversity efficiently and accurately. As such, they promise to be an important tool for biodiversity policy.

A second more fundamental aspect of biodiversity remains to be addressed in resource management. That is, how this diversity is actually connected on the landscape. It is these connections (e.g., herbivory, parasitism rates), not diversity itself, which ultimately dictates how these ecosystems function [65]). The DNA database we have created here will be important for revealing the mechanisms of how ecological systems work – a fact made more poignant by the cascade of economic consequences contingent upon budworm outbreaks. For instance, we anticipate that this library of DNA barcodes will permit the development of microarrays that permit the rapid field identification of species involved in the budworm food web – a tool desired by the monitoring agencies responsible for the management of the forests of Quebec and New Brunswick. Barcoding, when combined with long-term, ecologically relevant collections, promises the first rapid and efficient tool capable of delineating species interactions with unparalleled precision and speed. This type of research therefore promises to lead resource managers from a strictly population-based approach to a unified conceptual attack (i.e., from population to food web) on the major environmental issues facing society. The application of barcoding techniques within scientifically vigorous long-term monitoring campaigns will change the face of community ecology.

## Materials and Methods

### Field

For full description of the field methodologies and the collection localities see the Supporting Information file in [9]. In brief, six herbivore species (*Choristoneura fumiferana* (Clem), *Choristoneura rosaceana* (Harris), *Acleris variana* (Fernald), *Epinota radicana* (Heinrich), *Coleotechnites piceaella* (Kearfott), and *Coleotechnites atrupictella* (Deitz)) were sampled and reared from three forest plots that varied in degree of heterogeneity (i.e., % of plot basal area comprised of balsam fir (*Abies balsamea*)). The six herbivores selected were the species most frequently sampled during twenty plot-years of field collections. Although other herbivores were present (particularly in the most heterogeneous plot) these were rare. The supporting information files in [9] list all other cases where we could not associate parasitoids with their hosts. From these herbivore species, approximately 100 different parasitoid species were reared, identified morphologically with the best keys and collaboration with the appropriate taxonomic expertise at the Canadian National Collection of Insects in Ottawa, Ontario, Canada. Specimens were selected for DNA barcoding from as broad a series of representative hosts as was evident from the rearing program. While New Brunswick has historically treated areas of budworm outbreak with pesticides, the three plots sampled in this work were not subject to such pesticide treatments.

### Molecular

DNA extracts were prepared from single legs using a glass fibre protocol [66]. Extracts were re-suspended in 30 µl of dH2O, and a 658-bp region near the 5’ terminus of the COI gene was amplified using primers (LepF1–LepR1) following standard protocols [15], [16], [17]. Composite sequences were generated using internal primers when initial amplification was not successful[15], [16], [17]. Primer information for individual sequences can be retrieved from BOLD using the Process IDs detailed in Table S1, but primers are as detailed in [15]. Sequence divergences were calculated using the K2P distance model and a NJ tree of K2P distance was created to provide a graphic representation of the among-species divergences. Full details of methodology are as in [15], [16], [17]. All sequence data are available on BOLD (www.barcodinglife.org) in the public project: Spruce Budworm food web parasitoids and hosts [ASSPP]. All collection information, BOLD, and GenBank accessions for all sequences are listed in Table S1.

### MOTU

Each individual was assigned to a barcode cluster using barcode divergences of approximately 2% in BOLD. Annotations to these MOTU assignments were considered based on data from alternative markers, and/or ecological information (see Figure S1). MOTU identification results were compared with the species delineated by a comprehensive morphological study.

When barcode analyses suggested morphologically cryptic mitochondrial diversity we amplified portions of the rRNA gene region 1) for a portion of the large subunit (LSU or 28S – the variable D2 region) – 593 individuals, or 2), the internal transcribed spacer region 1 (80 individuals) or 2 (93 individuals) in addition to the CO1 barcode region. rDNA sequences facilitated our interpretation of morphologically cryptic and geographically sympatric deep mtDNA splits (correlated splitting within the independent rDNA sequence supports the hypothesis of morphologically cryptic species, while the lack of a split suggests mtDNA variation within a species).

Within the variable D2 region of 28S, the forward primer corresponds to positions 3549–3568 in *Drosophila melanogaster* reference sequence (Genbank M21017) while the ITS1 sequence was generated using primers where the forward primer corresponds to positions 1822–1843 in the same reference sequence. GenBank accessions for all complementary marker sequences are in Table S1.

The ITS2 region has been proposed as, and discussed as, a potential, “holy grail” for species determination [32], [34]. We compared the resolution of this gene region for species determination to morphologically defined or barcode defined species. To do so, we used the pipeline for ITS2 and complementary base pair determination described in Shuctlz et al [50]. Briefly, sequences were amplified using previously published primers [67] with the forward primer corresponds to positions 2805–2830 in *D. melanogaster* reference sequence (Genbank M21017). These were cleaned and trimmed using Sequencher (v4.5) and then imported into the ITS2 Database [68]. Here, if no significant matches were found in a structure search we input the sequences into 4SALE [69] where the program RNAFold was used to predict secondary structures. From these secondary structures, predictions were made regarding the presence of compensatory base changes (CBCs). These matrices were compared on a pairwise basis to other genetic discontinuities observed.

### PD

Neighbor-joining trees were constructed with MEGA (v4 [70]) using pairwise deletion and p-distance on all CO1 sequences greater than 200 bp (as measured from the 5’ end of the barcoding region to ensure overlap) and being produced by species parasitizing budworm (and not restricted to other related microlepidoptera). These trees were analyzed for their complement of phylogenetic diversity (PD) as measured in Conserve IV (v1.4.0b2) [71], [72] for each year for each collection site.

The food web was visualized pre- and post-barcoding using the program FoodWeb3D [73].

## Supporting Information

Figure S1 All specimen Neighbour-Joining tree from BOLD with Sample ID, Host, and BIN number. Specimens identification remains as in Eveleigh et al [9] to emphasize the positive effect that barcoding can have on identifying clerical errors, misidentifications, problematic taxonomy and contamination. As corrections are made - these will be reflected in BOLD and GenBank.
(PDF)

Table S1 All specimen information (collection locality, date, host information), BOLD and GenBank accessions for all specimens and all genetic markers.
(XLS)

Table S2 Those presumed to be polyphagous species that became specialists after barcoding.
(XLS)

Table S3 CO1 MOTU groups and their degree of support from corroborative markers (28S-D2, ITS2).
(XLS)

Table S4 VanVeen ITS2 alignment/structure and CBC table from GenBank specimens: AJ309962-AJ309965.
(DOC)
