# Supplementary material for: Barcoding a Quantified Food Web: Crypsis, Concepts, Ecology and Hypotheses
Source: PLoS One. 2011 Jul 6;6(7):e14424. doi: 10.1371/journal.pone.0014424 (PMC3130735; doi:10.1371/journal.pone.0014424)
Supplement: Figure S1 — All specimen Neighbour-Joining tree from BOLD with Sample ID, Host, and BIN number. Specimens identification remains as in Eveleigh et al [9] to emphasize the positive effect that barcoding can have on identifying clerical errors, misidentifications, problematic taxonomy and contamination. As corrections are made - these will be reflected in BOLD and GenBank. (PDF) [file pone.0014424.s001.pdf]

# BOLD TaxonID Tree

| | | |
|---|---|---|
| Project | : | Spruce Budworm food web parasitoids and hosts [ASSPP] |
| Date | : | 17-August-2010 |
| Data Type | : | Nucleotide |
| Distance Model | : | Kimura 2 Parameter |
| Codon Positions | : | 1st, 2nd, 3rd |
| Labels | : | Extra Info, SampleID, BIN name |
| Colorization | : | |
| Sequence Count | : | 1489 |
| Species count | : | 91 |
| Genus count | : | 60 |
| Family count | : | 11 |
| Unidentified | : | 3 |
| BIN Count | : | 111 |

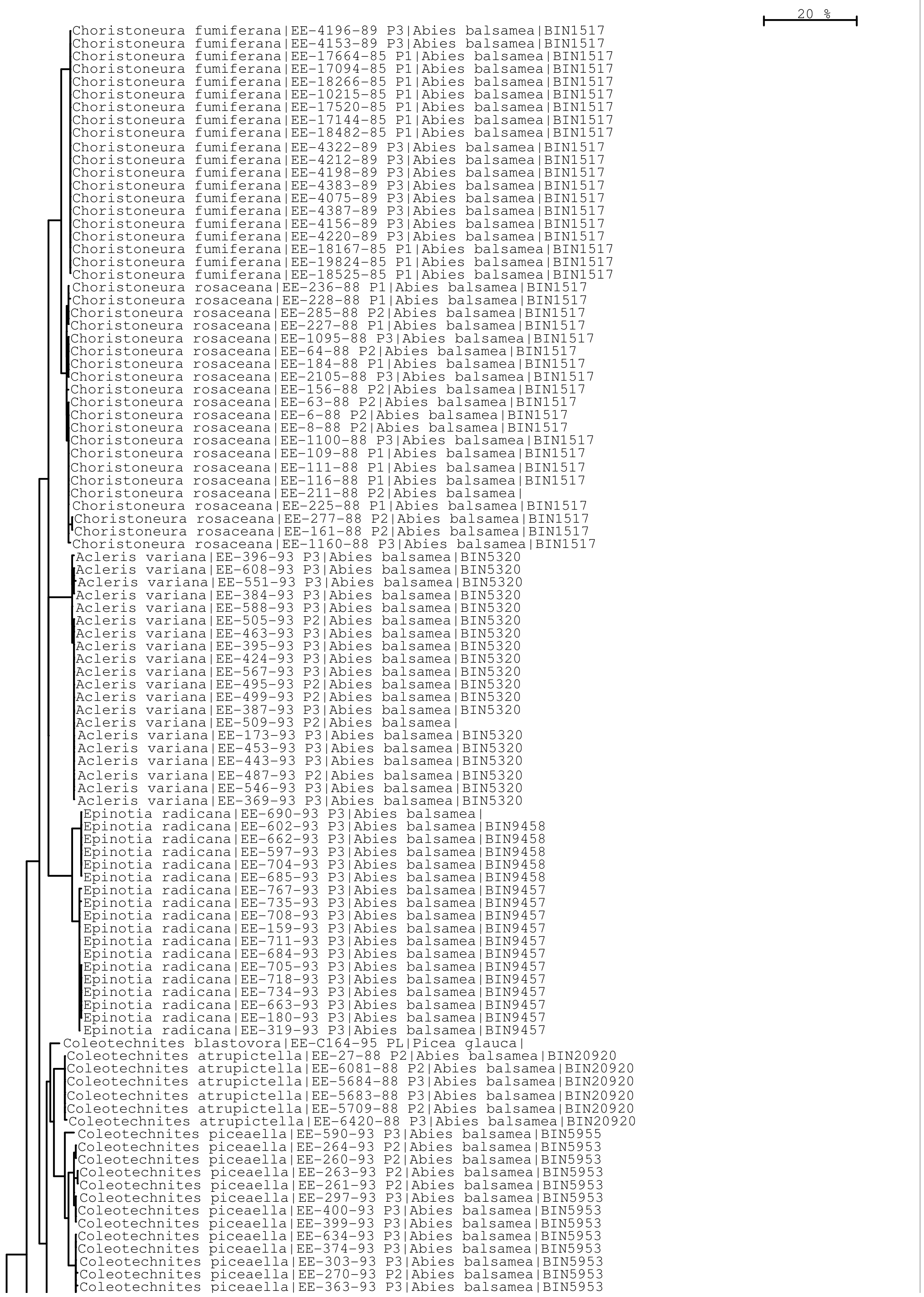
20 %
Choristoneura fumiferana|EE-4196-89 P3|Abies balsamea|BIN1517
Choristoneura fumiferana|EE-4153-89 P3|Abies balsamea|BIN1517
Choristoneura fumiferana|EE-17664-85 P1|Abies balsamea|BIN1517
Choristoneura fumiferana|EE-17094-85 P1|Abies balsamea|BIN1517
Choristoneura fumiferana|EE-18266-85 P1|Abies balsamea|BIN1517
Choristoneura fumiferana|EE-10215-85 P1|Abies balsamea|BIN1517
Choristoneura fumiferana|EE-17520-85 P1|Abies balsamea|BIN1517
Choristoneura fumiferana|EE-17144-85 P1|Abies balsamea|BIN1517
Choristoneura fumiferana|EE-18482-85 P1|Abies balsamea|BIN1517
Choristoneura fumiferana|EE-4322-89 P3|Abies balsamea|BIN1517
Choristoneura fumiferana|EE-4212-89 P3|Abies balsamea|BIN1517
Choristoneura fumiferana|EE-4198-89 P3|Abies balsamea|BIN1517
Choristoneura fumiferana|EE-4383-89 P3|Abies balsamea|BIN1517
Choristoneura fumiferana|EE-4075-89 P3|Abies balsamea|BIN1517
Choristoneura fumiferana|EE-4387-89 P3|Abies balsamea|BIN1517
Choristoneura fumiferana|EE-4156-89 P3|Abies balsamea|BIN1517
Choristoneura fumiferana|EE-4220-89 P3|Abies balsamea|BIN1517
Choristoneura fumiferana|EE-18167-85 P1|Abies balsamea|BIN1517
Choristoneura fumiferana|EE-19824-85 P1|Abies balsamea|BIN1517
Choristoneura fumiferana|EE-18525-85 P1|Abies balsamea|BIN1517
Choristoneura rosaceana|EE-236-88 P1|Abies balsamea|BIN1517
Choristoneura rosaceana|EE-228-88 P1|Abies balsamea|BIN1517
Choristoneura rosaceana|EE-285-88 P2|Abies balsamea|BIN1517
Choristoneura rosaceana|EE-227-88 P1|Abies balsamea|BIN1517
Choristoneura rosaceana|EE-1095-88 P3|Abies balsamea|BIN1517
Choristoneura rosaceana|EE-64-88 P2|Abies balsamea|BIN1517
Choristoneura rosaceana|EE-184-88 P1|Abies balsamea|BIN1517
Choristoneura rosaceana|EE-2105-88 P3|Abies balsamea|BIN1517
Choristoneura rosaceana|EE-156-88 P2|Abies balsamea|BIN1517
Choristoneura rosaceana|EE-63-88 P2|Abies balsamea|BIN1517
Choristoneura rosaceana|EE-6-88 P2|Abies balsamea|BIN1517
Choristoneura rosaceana|EE-8-88 P2|Abies balsamea|BIN1517
Choristoneura rosaceana|EE-1100-88 P3|Abies balsamea|BIN1517
Choristoneura rosaceana|EE-109-88 P1|Abies balsamea|BIN1517
Choristoneura rosaceana|EE-111-88 P1|Abies balsamea|BIN1517
Choristoneura rosaceana|EE-116-88 P1|Abies balsamea|BIN1517
Choristoneura rosaceana|EE-211-88 P2|Abies balsamea|
Choristoneura rosaceana|EE-225-88 P1|Abies balsamea|BIN1517
Choristoneura rosaceana|EE-277-88 P2|Abies balsamea|BIN1517
Choristoneura rosaceana|EE-161-88 P2|Abies balsamea|BIN1517
Choristoneura rosaceana|EE-1160-88 P3|Abies balsamea|BIN1517
Acleris variana|EE-396-93 P3|Abies balsamea|BIN5320
Acleris variana|EE-608-93 P3|Abies balsamea|BIN5320
Acleris variana|EE-551-93 P3|Abies balsamea|BIN5320
Acleris variana|EE-384-93 P3|Abies balsamea|BIN5320
Acleris variana|EE-588-93 P3|Abies balsamea|BIN5320
Acleris variana|EE-505-93 P2|Abies balsamea|BIN5320
Acleris variana|EE-463-93 P3|Abies balsamea|BIN5320
Acleris variana|EE-395-93 P3|Abies balsamea|BIN5320
Acleris variana|EE-424-93 P3|Abies balsamea|BIN5320
Acleris variana|EE-567-93 P3|Abies balsamea|BIN5320
Acleris variana|EE-495-93 P2|Abies balsamea|BIN5320
Acleris variana|EE-499-93 P2|Abies balsamea|BIN5320
Acleris variana|EE-387-93 P3|Abies balsamea|BIN5320
Acleris variana|EE-509-93 P2|Abies balsamea|
Acleris variana|EE-173-93 P3|Abies balsamea|BIN5320
Acleris variana|EE-453-93 P3|Abies balsamea|BIN5320
Acleris variana|EE-443-93 P3|Abies balsamea|BIN5320
Acleris variana|EE-487-93 P2|Abies balsamea|BIN5320
Acleris variana|EE-546-93 P3|Abies balsamea|BIN5320
Acleris variana|EE-369-93 P3|Abies balsamea|BIN5320
Epinotia radicana|EE-690-93 P3|Abies balsamea|
Epinotia radicana|EE-602-93 P3|Abies balsamea|BIN9458
Epinotia radicana|EE-662-93 P3|Abies balsamea|BIN9458
Epinotia radicana|EE-597-93 P3|Abies balsamea|BIN9458
Epinotia radicana|EE-704-93 P3|Abies balsamea|BIN9458
Epinotia radicana|EE-685-93 P3|Abies balsamea|BIN9458
Epinotia radicana|EE-767-93 P3|Abies balsamea|BIN9457
Epinotia radicana|EE-735-93 P3|Abies balsamea|BIN9457
Epinotia radicana|EE-708-93 P3|Abies balsamea|BIN9457
Epinotia radicana|EE-159-93 P3|Abies balsamea|BIN9457
Epinotia radicana|EE-711-93 P3|Abies balsamea|BIN9457
Epinotia radicana|EE-684-93 P3|Abies balsamea|BIN9457
Epinotia radicana|EE-705-93 P3|Abies balsamea|BIN9457
Epinotia radicana|EE-718-93 P3|Abies balsamea|BIN9457
Epinotia radicana|EE-734-93 P3|Abies balsamea|BIN9457
Epinotia radicana|EE-663-93 P3|Abies balsamea|BIN9457
Epinotia radicana|EE-180-93 P3|Abies balsamea|BIN9457
Epinotia radicana|EE-319-93 P3|Abies balsamea|BIN9457
Coleotechnites blastovora|EE-C164-95 PL|Picea glauca|
Coleotechnites atrupictella|EE-27-88 P2|Abies balsamea|BIN20920
Coleotechnites atrupictella|EE-6081-88 P2|Abies balsamea|BIN20920
Coleotechnites atrupictella|EE-5684-88 P3|Abies balsamea|BIN20920
Coleotechnites atrupictella|EE-5683-88 P3|Abies balsamea|BIN20920
Coleotechnites atrupictella|EE-5709-88 P2|Abies balsamea|BIN20920
Coleotechnites atrupictella|EE-6420-88 P3|Abies balsamea|BIN20920
Coleotechnites piceaella|EE-590-93 P3|Abies balsamea|BIN5955
Coleotechnites piceaella|EE-264-93 P2|Abies balsamea|BIN5953
Coleotechnites piceaella|EE-260-93 P2|Abies balsamea|BIN5953
Coleotechnites piceaella|EE-263-93 P2|Abies balsamea|BIN5953
Coleotechnites piceaella|EE-261-93 P2|Abies balsamea|BIN5953
Coleotechnites piceaella|EE-297-93 P3|Abies balsamea|BIN5953
Coleotechnites piceaella|EE-400-93 P3|Abies balsamea|BIN5953
Coleotechnites piceaella|EE-399-93 P3|Abies balsamea|BIN5953
Coleotechnites piceaella|EE-634-93 P3|Abies balsamea|BIN5953
Coleotechnites piceaella|EE-374-93 P3|Abies balsamea|BIN5953
Coleotechnites piceaella|EE-303-93 P3|Abies balsamea|BIN5953
Coleotechnites piceaella|EE-270-93 P2|Abies balsamea|BIN5953
Coleotechnites piceaella|EE-363-93 P3|Abies balsamea|BIN5953

Coleotechnites piceaella|EE-303-93 P3|Abies balsamea|BIN5953
Coleotechnites piceaella|EE-270-93 P2|Abies balsamea|BIN5953
Coleotechnites piceaella|EE-363-93 P3|Abies balsamea|BIN5953
Coleotechnites piceaella|EE-393-93 P3|Abies balsamea|BIN5953
Coleotechnites piceaella|EE-355-93 P3|Abies balsamea|BIN5953
Coleotechnites piceaella|EE-667-93 P3|Abies balsamea|BIN5953
Coleotechnites piceaella|EE-61-93 P3|Abies balsamea|BIN5953
Coleotechnites piceaella|EE-305-93 P3|Abies balsamea|BIN5953
Coleotechnites piceaella|EE-725-93 P3|Abies balsamea|BIN5953
Coleotechnites piceaella|EE-466-93 P3|Abies balsamea|BIN5953
Copidosoma sp.|EE-6491-87 P1|Coleotechnites piceaella|BIN6058
Elachertus sp.|EE-162-94 MP|Choristoneura fumiferana|BIN6058
Glypta sp.|EE-3094-85 P1|Choristoneura rosaceana|
Actia interrupta|EE-873-88 MP|Choristoneura fumiferana|BIN18437
Actia interrupta|EE-4096-90 P3|Choristoneura fumiferana|BIN18437
Actia interrupta|EE-4199a-90 P3|Choristoneura fumiferana|BIN18437
Actia interrupta|EE-4154-90 P3|Choristoneura fumiferana|BIN18437
Actia interrupta|EE-4548-89 P3|Choristoneura fumiferana|BIN18437
Actia interrupta|EE-4516-89 P3|Choristoneura fumiferana|BIN18437
Actia interrupta|EE-4132-89 P3|Choristoneura fumiferana|BIN18437
Actia interrupta|EE-4110-89 P3|Choristoneura fumiferana|BIN18437
Actia interrupta|EE-4057-89 P3|Choristoneura fumiferana|BIN18437
Actia interrupta|EE-4546-89 P3|Choristoneura fumiferana|BIN18437
Actia interrupta|EE-4558-89 P3|Choristoneura fumiferana|BIN18437
Actia interrupta|EE-752-88 MP|Choristoneura fumiferana|BIN18437
Actia interrupta|EE-994-88 MP|Choristoneura fumiferana|BIN18437
Actia interrupta|EE-870-88 MP|Choristoneura fumiferana|BIN18437
Actia interrupta|EE-882-88 MP|Choristoneura fumiferana|BIN18437
Lypha fumipennis|EE-5-83 P1|Choristoneura fumiferana|BIN56259
Lypha fumipennis|EE-6-83 P1|Choristoneura fumiferana|BIN56259
Lypha fumipennis|EE-3-83 P1|Choristoneura fumiferana|BIN56259
Ceromasia aurifons|EE-3515-92R|Choristoneura fumiferana|BIN62173
Eumea caesar|EE-16817-85 P1|Choristoneura fumiferana|BIN21602
Eumea caesar|EE-4698-91 P3|Choristoneura fumiferana|BIN21602
Eumea caesar|EE-5783-91 P3|Choristoneura fumiferana|BIN21602
Eumea caesar|EE-4606-91 P3|Choristoneura fumiferana|BIN21602
Eumea caesar|EE-3742-90 P3|Choristoneura fumiferana|BIN21602
Eumea caesar|EE-3781-90 P3|Choristoneura fumiferana|BIN21602
Eumea caesar|EE-3582-90 P3|Choristoneura fumiferana|BIN21602
Eumea caesar|EE-3164-90 P3|Choristoneura fumiferana|BIN21602
Eumea caesar|EE-3707-90 P3|Choristoneura fumiferana|BIN21602
Eumea caesar|EE-20-83 P1|Choristoneura fumiferana|BIN21602
Eumea caesar|EE-3839-89 P3|Choristoneura fumiferana|BIN21602
Eumea caesar|EE-19a-83 P1|Choristoneura fumiferana|BIN21602
Eumea caesar|EE-19b-83 P1|Choristoneura fumiferana|
Eumea caesar|EE-21-83 P1|Choristoneura fumiferana|BIN21602
Madremyia saundersii|EE-3738-90 P3|Choristoneura fumiferana|BIN107497
Madremyia saundersii|EE-22a-83 P1|Choristoneura fumiferana|
Phryxe pecosensis|EE-4306-89 P3|Choristoneura fumiferana|BIN21603
Phryxe pecosensis|EE-3324-90 P3|Choristoneura fumiferana|BIN21603
Phryxe pecosensis|EE-3966-90 P3|Choristoneura fumiferana|BIN21603
Phryxe pecosensis|EE-3936-90 P3|Choristoneura fumiferana|BIN21603
Phryxe pecosensis|EE-3821-90 P3|Choristoneura fumiferana|BIN21603
Phryxe pecosensis|EE-3726-90 P3|Choristoneura fumiferana|BIN21603
Phryxe pecosensis|EE-3123-90 P3|Choristoneura fumiferana|BIN21603
Phryxe pecosensis|EE-4268-89 P3|Choristoneura fumiferana|BIN21603
Phryxe pecosensis|EE-4247-89 P3|Choristoneura fumiferana|BIN21603
Eumea caesar|EE-4827-91 P3|Choristoneura fumiferana|BIN21603
Phryxe pecosensis|EE-3891-89 P3|Choristoneura fumiferana|BIN21603
Phryxe pecosensis|EE-15397-85 P1|Choristoneura fumiferana|
Phryxe pecosensis|EE-15623-85 P1|Choristoneura fumiferana|
Phryxe pecosensis|EE-15291-85 P1|Choristoneura fumiferana|
Phryxe pecosensis|EE-16123a-85 P1|Choristoneura fumiferana|
Smidtia fumiferanae|EE-2799-92 P3|Choristoneura fumiferana|
Agria affinis|EE-1-84 P1|Choristoneura fumiferana|BIN25699
Agria affinis|EE-5-84 P1|Choristoneura fumiferana|BIN25699
Agria affinis|EE-8-83 P1|Choristoneura fumiferana|BIN25699
Agria affinis|EE-2-84 P1|Choristoneura fumiferana|BIN25699
Agria affinis|EE-18-83 P1|Choristoneura fumiferana|BIN25699
Agria affinis|EE-3-84 P1|Choristoneura fumiferana|BIN25699
Agria affinis|EE-4-84 P1|Choristoneura fumiferana|BIN25699
Smidtia fumiferanae|EE-16-84 P1|Choristoneura fumiferana|BIN15914
Smidtia fumiferanae|EE-17-83 P1|Choristoneura fumiferana|BIN15914
Smidtia fumiferanae|EE-15-83 P1|Choristoneura fumiferana|BIN15914
Smidtia fumiferanae|EE-6-84 P1|Choristoneura fumiferana|BIN15914
Smidtia fumiferanae|EE-14-83 P1|Choristoneura fumiferana|BIN15914
Smidtia fumiferanae|EE-12-83 P1|Choristoneura fumiferana|BIN15914
Smidtia fumiferanae|EE-11-83 P1|Choristoneura fumiferana|
Smidtia fumiferanae|EE-9-83 P1|Choristoneura fumiferana|BIN15914
Smidtia fumiferanae|EE-13-83-P1|Choristoneura fumiferana|BIN15914
Smidtia fumiferanae|EE-7-83 P1|Choristoneura fumiferana|BIN15914
Smidtia fumiferanae|EE-3920-89 P3|Choristoneura fumiferana|BIN15914
Smidtia fumiferanae|EE-4024a-89 P3|Choristoneura fumiferana|BIN15914
Smidtia fumiferanae|EE-4118-89 P3|Choristoneura fumiferana|BIN15914
Smidtia fumiferanae|EE-4120-89 P3|Choristoneura fumiferana|BIN15914
Smidtia fumiferanae|EE-5476-91 P3|Choristoneura fumiferana|BIN15914
Smidtia fumiferanae|EE-5521-91 P3|Choristoneura fumiferana|BIN15914
Smidtia fumiferanae|EE-5626-91 P3|Choristoneura fumiferana|BIN15914
Smidtia fumiferanae|EE-5462-91 P3|Choristoneura fumiferana|BIN15914
Smidtia fumiferanae|EE-5863-88 P3|Choristoneura fumiferana|BIN15914
Smidtia fumiferanae|EE-4024b-89 P3|Choristoneura fumiferana|BIN15914
Mesopolobus verditer|EE-282-86 P1|Glypta fumiferanae|BIN4053
Mesopolobus verditer|EE-13469-86 P1|Choristoneura fumiferana|BIN4053
Mesopolobus verditer|EE-6748-88 P3|Choristoneura fumiferana|BIN4053
Mesopolobus verditer|EE-13504i-86 P2|Choristoneura fumiferana|BIN4053
Mesopolobus verditer|EE-257-86 P2|Glypta fumiferanae|BIN4053
Mesopolobus verditer|EE-50-86 P2|Apanteles fumiferanae|BIN4053
Mesopolobus verditer|EE-706-86 P1|Meteorus trachynotus|BIN4053
Mesopolobus verditer|EE-844-86 P2|Meteorus trachynotus|BIN4053
Mesopolobus verditer|EE-1134ii-91 P3|Nemorilla pyste|BIN4053
Mesopolobus verditer|EE-306-88 P3|Meteorus trachynotus|BIN4053
Mesopolobus verditer|EE-12810-86 P2|Choristoneura fumiferana|BIN4053
[illegible]

Mesopolobus verditer|EE-11341i-91 P3|Nemorilla pyste|BIN4053
Mesopolobus verditer|EE-306-88 P3|Meteorus trachynotus|BIN4053
Mesopolobus verditer|EE-12810-86 P2|Choristoneura fumiferana|BIN4053
Mesopolobus verditer|EE-410-90 P3|Glypta fumiferanae|BIN4053
Mesopolobus verditer|EE-444-87 P2|Meteorus trachynotus|BIN4053
Mesopolobus verditer|EE-1173-88 P3|Apanteles fumiferanae|BIN4053
Mesopolobus verditer|EE-373-86 P2|Glypta fumiferanae|BIN4053
Mesopolobus verditer|EE-134-86 P2|Meteorus trachynotus|BIN4053
Mesopolobus verditer|EE-13486-86 P2|Choristoneura fumiferana|BIN4053
Mesopolobus verditer|EE-12728-86 P1|Choristoneura fumiferana|BIN4053
Mesopolobus verditer|EE-6775-88 P3|Choristoneura fumiferana|BIN4053
Mesopolobus verditer|EE-303-86 P2|Glypta fumiferanae|BIN4053
Mesopolobus verditer|EE-6649-88 P3|Choristoneura fumiferana|BIN4053
Mesopolobus verditer|EE-13510-86 P1|Coleotechnites blastovora|BIN4053
Mesopolobus verditer|EE-1279-89 P3|Meteorus trachynotus|BIN4053
Mesopolobus verditer|EE-68-86 P2|Apanteles fumiferanae|
Mesopolobus verditer|EE-1114-88 P3|Dolichogenidea absona|BIN4053
Mesopolobus verditer|EE-6446-88 P3|Choristoneura fumiferana|BIN4053
Mesopolobus verditer|EE-12903-86 P1|Choristoneura fumiferana|BIN4053
Mesopolobus verditer|EE-1353-91 P3|Eumea caesar|BIN4053
Mesopolobus verditer|EE-672-85 P1|Glypta fumiferanae|BIN4053
Mesopolobus verditer|EE-13365-86 P1|Choristoneura fumiferana|BIN4053
Mesopolobus verditer|EE-665-86 P1|Apanteles fumiferanae|
Mesopolobus sp.|EE-99-85 P1|Apanteles fumiferanae|
Mesopolobus verditer|EE-13508-86 P1|Choristoneura fumiferana|BIN4053
Mesopolobus verditer|EE-764-86 P2|Glypta fumiferanae|BIN4053
Mesopolobus verditer|EE-759-86 P2|Glypta fumiferanae|BIN4053
Mesopolobus verditer|EE-13249-86 P2|Choristoneura fumiferana|BIN4053
Mesopolobus verditer|EE-879-86 P2|Glypta fumiferanae|BIN4053
Mesopolobus verditer|EE-12851-86 P2|Choristoneura fumiferana|
Mesopolobus verditer|EE-646-86 P1|Glypta fumiferanae|
Mesopolobus verditer|EE-13525-86 P1|Choristoneura fumiferana|BIN4053
Mesopolobus verditer|EE-13317-86 P2|Choristoneura fumiferana|BIN4053
Mesopolobus verditer|EE-13698i-86 P1|Epinota radicana|BIN4053
Mesopolobus verditer|EE-13698ii-86 P1|Epinota radicana|BIN4053
Mesopolobus verditer|EE-13698iii-86 P1|Epinota radicana|BIN4053
Mesopolobus verditer|EE-13698iv-86 P1|Epinota radicana|BIN4053
Mesopolobus verditer|EE-223-86 P1|Glypta fumiferanae|
Mesopolobus verditer|EE-654-90 P3|Glyptapanteles sp|BIN4053
Mesopolobus verditer|EE-176-86 P1|Apanteles fumiferanae|
Mesopolobus verditer|EE-1134i-91 P3|Nemorilla pyste|BIN4053
Mesopolobus verditer|EE-1374-89 P3|Meteorus trachynotus|BIN4053
Mesopolobus verditer|EE-6864-88 P3|Choristoneura fumiferana|BIN4053
Mesopolobus verditer|EE-6701-88 P3|Choristoneura fumiferana|BIN4053
Mesopolobus verditer|EE-12462-86 P2|Choristoneura fumiferana|BIN4053
Mesopolobus verditer|EE-13021-86 P2|Choristoneura fumiferana|BIN4053
Mesopolobus verditer|EE-144-86 P2|Apanteles fumiferanae|BIN4053
Mesopolobus verditer|EE-143-86 P2|Apanteles fumiferanae|BIN4053
Mesopolobus verditer|EE-140-86 P2|Apanteles fumiferanae|BIN4053
Mesopolobus verditer|EE-351-87 P2|Meteorus trachynotus|BIN4053
Mesopolobus verditer|EE-13230-86 P2|Choristoneura fumiferana|BIN4053
Mesopolobus tortricis|EE-12793-86 P2|Choristoneura fumiferanae|BIN4053
Mesopolobus verditer|EE-1148-91 P3|Nemorilla pyste|BIN4053
Mesopolobus verditer|EE-13323-86 P1|Choristoneura fumiferana|BIN4053
Mesopolobus verditer|EE-13400-86 P1|Choristoneura fumiferana|BIN4053
Mesopolobus verditer|EE-6591-88 P3|Choristoneura fumiferana|BIN4053
Mesopolobus verditer|EE-30-87 P1|Apanteles fumiferanae|BIN4053
Mesopolobus verditer|EE-6751-88 P3|Choristoneura fumiferana|BIN4053
Mesopolobus verditer|EE-13055-86 P2|Choristoneura fumiferana|BIN4053
Mesopolobus verditer|EE-6883-88 P3|Choristoneura fumiferana|BIN4053
Mesopolobus verditer|EE-233-86 P1|Apanteles fumiferanae|BIN4053
Mesopolobus verditer|EE-6727-88 P3|Choristoneura fumiferana|BIN4053
Mesopolobus verditer|EE-380-86 P2|Meteorus trachynotus via Glypta fumiferanae|BIN4053
Mesopolobus verditer|EE-1212-91 P3|Eumea caesar|BIN4053
Mesopolobus verditer|EE-231ii-87 P2|Glyptapanteles sp|BIN4053
Mesopolobus verditer|EE-331-87 P2|Meteorus trachynotus|BIN4053
Mesopolobus verditer|EE-660-85 P1|Glypta fumiferanae|BIN4053
Mesopolobus verditer|EE-69-86 P2|Apanteles fumiferanae|BIN4053
Mesopolobus verditer|EE-482-86 P2|Glypta fumiferanae|BIN4053
Mesopolobus verditer|EE-491-87 P2|Stictopisthus sp. via Apanteles fumiferanae|BIN4053
Mesopolobus verditer|EE-12673-86 P1|Choristoneura fumiferana|BIN4053
Mesopolobus verditer|EE-13309i-86 P2|Choristoneura fumiferana|BIN4053
Mesopolobus verditer|EE-1249-89 P3|Meteorus trachynotus|BIN4053
Mesopolobus verditer|EE-1158-88 P3|Apanteles fumiferanae|BIN4053
Mesopolobus verditer|EE-319-86 P1|Scambus sp. via Glypta fumiferanae|BIN4053
Mesopolobus verditer|EE-417-87 P2|Meteorus trachynotus|BIN4053
Mesopolobus verditer|EE-13093-86 P1|Choristoneura fumiferana|BIN4053
Mesopolobus verditer|EE-15-87 P1|Apanteles fumiferanae|BIN4053
Mesopolobus verditer|EE-1186-88 P3|Glypta fumiferanae|BIN4053
Mesopolobus verditer|EE-95-86 P2|Apanteles fumiferanae|BIN4053
Mesopolobus verditer|EE-258-88 P3|Apanteles fumiferanae|BIN4053
Mesopolobus verditer|EE-52-87 P1|Apanteles fumiferanae|BIN4053
Mesopolobus verditer|EE-139-88 P3|Apanteles fumiferanae|BIN4055
Mesopolobus verditer|EE-40-95 MP|Dolichogenidea absona|BIN4055
Mesopolobus verditer|EE-19-95 MP|Dolichogenidea absona|BIN4055
Mesopolobus verditer|EE-20-95 MP|Dolichogenidea absona|BIN4055
Mesopolobus verditer|EE-152-88 P3|Apanteles fumiferanae|BIN4055
Mesopolobus verditer|EE-327-88 P3|Meteorus trachynotus|BIN4055
Mesopolobus verditer|EE-271-88 P3|Meteorus trachynotus|BIN4055
Mesopolobus verditer|EE-267-88 P3|Meteorus trachynotus|BIN4055
Mesopolobus verditer|EE-226-88 P3|Apanteles fumiferanae|
Mesopolobus verditer|EE-86-88 P3|Apanteles fumiferanae|
Mesopolobus verditer|EE-93-88 P3|Apanteles fumiferanae|
Mesopolobus verditer|EE-265-88 P3|Apanteles fumiferanae|
Mesopolobus verditer|EE-18-95 MP|Dolichogenidea absona|
Mesopolobus verditer|EE-23-89 MP|Dolichogenidea absona|BIN4055
Mesopolobus tortricis|EE-6675-88 P3|Choristoneura fumiferanae|BIN11881
Pteromalus phycidis|EE-552-87 P2|Meteorus trachynotus|BIN8292
Pteromalus phycidis|EE-1192-86 P2|Meteorus trachynotus|
Pteromalus phycidis|EE-60-88 MP|Apanteles morrisi|BIN8290
Pteromalus phycidis|EE-285-88 P3|Apanteles fumiferanae|
Pteromalus phycidis|EE-304-88 P3|Apanteles fumiferanae|BIN8290
Pteromalus phycidis|EE-32-89 MP|Apanteles sp.|BIN4054

Pteromalus phycidis|EE-285-88 P3|Apanteles fumiferanae|
Pteromalus phycidis|EE-304-88 P3|Apanteles fumiferanae|BIN8290
Pteromalus phycidis|EE-32-89 MP|Apanteles sp.|BIN4054
Pteromalus phycidis|EE-37-92 MP|Apanteles petrovae|BIN4054
Mesopolobus verditer|EE-1137-93 MP|Coleotechnites piceaella|BIN4054
Pteromalus phycidis|EE-159-89 MP|Apanteles sp.|BIN4054
Pteromalus phycidis|EE-71-93 MP|Dolichogenidea absona|BIN4054
Pteromalus phycidis|EE-25-90 MP|Dolichogenidea absona|BIN4054
Copidosoma sp.|EE-6569-88 P2|Coleotechnites piceaella|
Copidosoma sp.|EE-4205-87 P2|Coleotechnites piceaella|
Copidosoma sp.|EE-7063-87 P1|Coleotechnites piceaella|
Copidosoma sp.|EE-10579-86 P1|Coleotechnites piceaella|
Copidosoma sp.|EE-180-89 MP|Coleotechnites piceaella|
Copidosoma sp.|EE-6419-87 P1|Coleotechnites piceaella|
Copidosoma sp.|EE-4423-88 P2|Coleotechnites piceaella|
Copidosoma sp.|EE-5712-88 P2|Coleotechnites piceaella|
Copidosoma sp.|EE-4046-86 P1|Coleotechnites piceaella|
Copidosoma sp.|EE-5062-87 P2|Coleotechnites piceaella|BIN14285
Copidosoma sp.|EE-1263-87 P1|Coleotechnites piceaella|BIN14285
Copidosoma sp.|EE-5897-86 P1|Coleotechnites piceaella|BIN14285
Copidosoma sp.|EE-333-88 P2|Coleotechnites piceaella|BIN14285
Copidosoma sp.|EE-4368-90 P3|Coleotechnites piceaella|BIN14285
Copidosoma sp.|EE-9049-86 P1|Coleotechnites piceaella|BIN14285
Copidosoma sp.|EE-2216-90 P3|Coleotechnites piceaella|BIN14285
Copidosoma sp.|EE-12-90 P3|Coleotechnites piceaella|BIN14285
Copidosoma sp.|EE-3518-91 P3|Coleotechnites piceaella|BIN14285
Copidosoma sp.|EE-7728-87 P2|Coleotechnites piceaella|BIN14285
Copidosoma sp.|EE-6468-87 P1|Coleotechnites piceaella|BIN14285
Copidosoma sp.|EE-2538-92 P3|Coleotechnites piceaella|BIN14285
Copidosoma sp.|EE-6657-87 P2|Coleotechnites piceaella|BIN14285
Copidosoma sp.|EE-5436-88 P2|Coleotechnites piceaella|BIN14285
Copidosoma sp.|EE-2277-90 P3|Coleotechnites piceaella|
Copidosoma sp.|EE-3425-91 P3|Coleotechnites piceaella|BIN14285
Copidosoma sp.|EE-2795-90 P2|Coleotechnites piceaella|BIN14285
Copidosoma sp.|EE-327-88 P2|Coleotechnites piceaella|BIN14285
Copidosoma sp.|EE-2287-86 P1|Coleotechnites piceaella|BIN14285
Copidosoma sp.|EE-3377-91 P3|Coleotechnites piceaella|BIN14285
Pediobius crassicornis|EE-6549ii-91R P3|Psychophagus sp.|
Pediobius sp.|EE-13689ii-86 P1|Choristoneura fumiferanae|BIN15902
Pediobius sp.|EE-13321i-86 P2|Choristoneura fumiferanae|BIN15902
Baryscapus sp.|EE-13313i-86 P2|Choristoneura fumiferanae|BIN15902
Pediobius sp.|EE-13620ii-86 P1|Choristoneura fumiferanae|BIN15902
Pediobius sp.|EE-12995ii-86 P2|Choristoneura fumiferanae|BIN15902
Pediobius sp.|EE-12995i-86 P2|Choristoneura fumiferanae|
Pteromalus phycidis|EE-1084-85 P1|Glypta fumiferanae|BIN8291
Pteromalus phycidis|EE-6963-88 P3|Choristoneura fumiferana|BIN8289
Pteromalus phycidis|EE-396-85 P1|Apanteles fumiferanae|BIN8289
Pteromalus phycidis|EE-4365-90 P3|Choristoneura fumiferana|BIN8289
Pteromalus phycidis|EE-189-95 MP|Apanteles sp.|BIN8289
Pteromalus phycidis|EE-331-85 P1|Apanteles fumiferanae|BIN8289
Pteromalus phycidis|EE-201-88 P3|Apanteles fumiferanae|BIN8289
Pteromalus phycidis|EE-7017-88 P3|Choristoneura fumiferana|BIN8289
Pteromalus phycidis|EE-6894-88 P3|Choristoneura fumiferana|BIN8289
Pteromalus phycidis|EE-1293-91 P3|Glyptapanteles sp.|BIN8289
Pteromalus phycidis|EE-6780-88 P3|Choristoneura fumiferana|BIN8289
Pteromalus phycidis|EE-118-88 P3|Apanteles fumiferanae|BIN8289
Pteromalus phycidis|EE-90-85 P1|Apanteles fumiferanae|BIN8289
Pteromalus phycidis|EE-449-88 P3|Apanteles fumiferanae|BIN8289
Pteromalus phycidis|EE-4631-89 P3|Coleotechnites piceaella|BIN8289
Pteromalus phycidis|EE-344-85 P1|Apanteles fumiferanae|BIN8289
Pteromalus phycidis|EE-316-85 P1|Apanteles fumiferanae|BIN8289
Pteromalus phycidis|EE-292-88 P3|Apanteles fumiferanae|BIN8289
Pteromalus phycidis|EE-526-86 P1|Apanteles fumiferanae|BIN8289
Pteromalus phycidis|EE-4208-90 P3|Choristoneura fumiferana|BIN8289
Pteromalus phycidis|EE-7039-88 P3|Choristoneura fumiferana|BIN8289
Pteromalus phycidis|EE-6751i-88 P3|Choristoneura fumiferana|BIN8289
Pteromalus phycidis|EE-413-86 P1|Glypta fumiferanae|BIN8289
Pteromalus phycidis|EE-6950-88 P3|Choristoneura fumiferana|BIN8289
Pteromalus phycidis|EE-4399-90 P3|Choristoneura fumiferana|BIN8289
Pteromalus phycidis|EE-1366-88 P3|Meteorus trachynotus|BIN8289
Pteromalus phycidis|EE-114-85 P1|Apanteles fumiferanae|BIN8289
Pteromalus phycidis|EE-94-85 P1|Apanteles fumiferanae|BIN8289
Pteromalus phycidis|EE-56-93 MP|Dolichogenidea absona|BIN8289
Pteromalus phycidis|EE-26-90 MP|Dolichogenidea absona|BIN8289
Pteromalus phycidis|EE-22-90 MP|Dolichogenidea absona|BIN8289
Pteromalus phycidis|EE-15-90 MP|Dolichogenidea absona|BIN8289
Pteromalus phycidis|EE-16-90 MP|Dolichogenidea absona|BIN8289
Pteromalus phycidis|EE-124-85 P1|Apanteles fumiferanae|BIN8289
Elachertus sp.|EE-963-93 MP|Choristoneura fumiferana|
Elachertus sp.|EE-1019-93 MP|Choristoneura fumiferana|
Elachertus sp.|EE-550-91 MP|Choristoneura fumiferana|BIN49926
Elachertus sp.|EE-200-94 MP|Choristoneura fumiferana|BIN49926
Elachertus sp.|EE-1021-93 MP|Choristoneura fumiferana|
Elachertus sp.|EE-217-94 MP|Choristoneura fumiferana|BIN49926
Euplectrus maculiventris|EE-E33-94 P2|Choristoneura fumiferana|
Baryscapus coerulescens|EE-122-88 MP|Apanteles sp.|BIN4401
Baryscapus coerulescens|EE-41-88 MP|Apanteles morrisi|BIN4401
Baryscapus coerulescens|EE-382-95 MP|Elachertus cacoeciae|BIN4401
Baryscapus coerulescens|EE-34-88 MP|Apanteles morrisi|BIN4401
Baryscapus coerulescens|EE-111-88 MP|Apanteles morrisi|BIN4401
Baryscapus coerulescens|EE-286-85 P1|Apanteles fumiferanae|BIN4401
Baryscapus coerulescens|EE-130-88 MP|Apanteles morrisi|BIN4401
Baryscapus coerulescens|EE-133-86 P2|Apanteles fumiferanae|BIN4401
Baryscapus coerulescens|EE-324-85 P1|Apanteles fumiferanae|BIN4401
Baryscapus coerulescens|EE-152-85 P1|Apanteles fumiferanae|BIN4401
Baryscapus coerulescens|EE-132-88 MP|Dolichogenidea absona|BIN4401
Baryscapus coerulescens|EE-61-88 P3|Apanteles fumiferanae|BIN4401
Baryscapus coerulescens|EE-4883-89 P3|Choristoneura fumiferana|BIN4401
Baryscapus coerulescens|EE-246-95 MP|Apanteles sp.|BIN4401
Baryscapus coerulescens|EE-30-88 MP|Dolichogenidea absona|BIN4401
Baryscapus coerulescens|EE-145-88 P3|Apanteles fumiferanae|BIN4401
Baryscapus coerulescens|EE-23-94 MP|Elachertus cacoeciae|BIN4401
[illegible]

Baryscapus coerulescens|EE-30-88 MP|Dolichogenidea absona|BIN4401
Baryscapus coerulescens|EE-145-88 P3|Apanteles fumiferanae|BIN4401
Baryscapus coerulescens|EE-23-94 MP|Elachertus cacoeciae|BIN4401
Baryscapus coerulescens|EE-663-87 P2|Meteorus trachynotus|BIN4401
Baryscapus coerulescens|EE-40-88 MP|Apanteles morrisi|BIN4401
Baryscapus coerulescens|EE-70-88 MP|Apanteles morrisi|BIN4401
Baryscapus coerulescens|EE-102-85 P1|Apanteles fumiferanae|BIN4401
Baryscapus sp.|EE-12924ii-86 P1|Choristoneura fumiferanae|BIN4401
Baryscapus sp.|EE-12893ii-86 P1|Choristoneura fumiferanae|BIN4401
Baryscapus coerulescens|EE-174-88 P3|Apanteles fumiferanae|BIN4401
Baryscapus sp.|EE-12824-86 P2|Choristoneura fumiferanae|BIN4401
Baryscapus coerulescens|EE-166-86 P2|Apanteles fumiferanae|BIN4401
Baryscapus coerulescens|EE-4-88 P1|Dolichogenidea absona|BIN4401
Baryscapus coerulescens|EE-460-86 P2|Apanteles fumiferanae|BIN4401
Baryscapus coerulescens|EE-7066-88 P3|Choristoneura fumiferana|BIN4401
Baryscapus coerulescens|EE-1187-91 P3|Mesopolobus verditer via Glypta fumiferanae|BIN4401
Baryscapus coerulescens|EE-55-88 P3|Apanteles fumiferanae|BIN4401
Baryscapus coerulescens|EE-135-88 P3|Apanteles fumiferanae|BIN4401
Baryscapus coerulescens|EE-833-88 P3|Elasmus atratus via Apanteles fumiferanae|BIN4401
Baryscapus coerulescens|EE-661ii-87 P2|Mesopolobus verditer via Meteorus trachynotus|BIN4401
Baryscapus coerulescens|EE-194-88 MP|Apanteles morrisi|BIN4401
Baryscapus coerulescens|EE-131-88 P3|Apanteles fumiferanae|BIN4401
Baryscapus coerulescens|EE-58-88 P3|Apanteles fumiferanae|BIN4401
Baryscapus coerulescens|EE-147-88 P3|Apanteles fumiferanae|BIN4401
Baryscapus coerulescens|EE-215-86 P2|Apanteles fumiferanae|BIN4401
Baryscapus sp.|EE-3909-92 P3|Choristoneura fumiferanae|BIN4401
Baryscapus coerulescens|EE-12-88 MP|Dolichogenidea absona|BIN4401
Baryscapus coerulescens|EE-317-85 P1|Apanteles fumiferanae|BIN4401
Baryscapus coerulescens|EE-292-85 P1|Apanteles fumiferanae|BIN4401
Baryscapus coerulescens|EE-4-93 MP|Apanteles sp.|BIN4401
Baryscapus coerulescens|EE-39-91 MP|Apanteles morrisi|BIN4401
Baryscapus coerulescens|EE-9-88 P1|Apanteles petrovae|BIN4401
Baryscapus coerulescens|EE-167-95 MP|Apanteles sp.|BIN4401
Baryscapus coerulescens|EE-7020-88 P3|Choristoneura fumiferana|BIN4401
Baryscapus sp.|EE-13309ii-86 P2|Choristoneura fumiferana|BIN4401
Baryscapus sp.|EE-3842-92 P3|Choristoneura fumiferanae|BIN4401
Baryscapus coerulescens|EE-205-86 P2|Apanteles fumiferanae|BIN4401
Baryscapus coerulescens|EE-92-88 P3|Apanteles fumiferanae|BIN4401
Baryscapus coerulescens|EE-326-85 P1|Apanteles fumiferanae|BIN4401
Baryscapus coerulescens|EE-324-95 MP|Elachertus cacoeciae|BIN4401
Baryscapus coerulescens|EE-7088ii-88 P3|Choristoneura fumiferana|BIN4401
Baryscapus coerulescens|EE-7035-88 P3|Choristoneura fumiferana|BIN4401
Baryscapus coerulescens|EE-7088iii-88 P3|Choristoneura fumiferana|BIN4401
Baryscapus coerulescens|EE-20-88 MP|Dolichogenidea absona|BIN4401
Baryscapus coerulescens|EE-7088i-88 P3|Choristoneura fumiferana|BIN4401
Baryscapus coerulescens|EE-116-86 P2|Apanteles fumiferanae|BIN4401
Baryscapus coerulescens|EE-10-88 MP|Dolichogenidea absona|BIN4401
Baryscapus coerulescens|EE-18-88 MP|Dolichogenidea absona|BIN4401
Baryscapus coerulescens|EE-31-88 MP|Dolichogenidea absona|BIN4401
Baryscapus coerulescens|EE-26-88 MP|Dolichogenidea absona|BIN4401
Baryscapus coerulescens|EE-202-85 P1|Apanteles fumiferanae|BIN4401
Baryscapus coerulescens|EE-7088iv-88 P3|Choristoneura fumiferana|BIN4401
Baryscapus coerulescens|EE-400-90 P3|Mesopolobus sp. Via Apanteles fumiferanae|BIN4401
Baryscapus sp.|EE-13312-86 P2|Choristoneura fumiferanae|BIN4401
Baryscapus coerulescens|EE-1505-89 P3|Meteorus trachynotus|BIN4401
Baryscapus sp.|EE-7714ii-87 P1|Choristoneura fumiferanae|BIN4401
Baryscapus coerulescens|EE-285-85 P1|Apanteles fumiferanae|BIN4401
Baryscapus sp.|EE-12815ii-86 P2|Choristoneura fumiferanae|BIN4401
Baryscapus coerulescens|EE-67-93 MP|Apanteles sp.|BIN4401
Baryscapus coerulescens|EE-1314-89 P3|Mesopolobus verditer via Apanteles fumiferanae|BIN4401
Baryscapus coerulescens|EE-193-88 MP|Apanteles morrisi|BIN4401
Baryscapus coerulescens|EE-46-89 MP|Dolichogenidea absona|BIN4401
Baryscapus coerulescens|EE-293-85 P1|Apanteles fumiferanae|BIN4401
Baryscapus coerulescens|EE-1027-91 P3|Mesopolobus verditer via Glypta fumiferanae|BIN4401
Baryscapus coerulescens|EE-29-88 MP|Dolichogenidea absona|BIN4401
Baryscapus coerulescens|EE-7024-88 P3|Choristoneura fumiferana|BIN4401
Baryscapus coerulescens|EE-12-89 MP|Apanteles sp.|BIN4401
Baryscapus sp.|EE-13504ii-86 P2|Choristoneura fumiferana|
Baryscapus coerulescens|EE-535-91 P3|Mesopolobus sp. Via Glypta fumiferanae|BIN4401
Baryscapus coerulescens|EE-359-86 P2|Apanteles fumiferanae|BIN4401
Baryscapus coerulescens|EE-64-88 P3|Apanteles fumiferanae|BIN4401
Baryscapus coerulescens|EE-65-93 MP|Apanteles sp.|BIN4401
Baryscapus coerulescens|EE-32-93 MP|Apanteles sp.|BIN4401
Baryscapus sp.|EE-13308i-86 P2|Choristoneura fumiferanae|BIN4401
Baryscapus coerulescens|EE-7057-88 P3|Choristoneura fumiferana|BIN4401
Baryscapus coerulescens|EE-97-86 P2|Apanteles fumiferanae|BIN4401
Baryscapus sp.|EE-13577-86 P2|Choristoneura fumiferanae|BIN4401
Baryscapus sp.|EE-12813ii-86 P2|Choristoneura fumiferanae|BIN4401
Baryscapus coerulescens|EE-213-86 P2|Apanteles fumiferanae|BIN4401
Baryscapus sp.|EE-12796ii-86 P2|Choristoneura fumiferanae|BIN4401
Baryscapus coerulescens|EE-289-95 MP|Elachertus cacoeciae|BIN4403
Baryscapus coerulescens|EE-38-93 MP|Apanteles sp.|BIN4403
Baryscapus sp.|EE-7714i-87 P1|Choristoneura fumiferanae|BIN15901
Baryscapus sp.|EE-13042-86 P2|Choristoneura fumiferanae|BIN15901
Baryscapus sp.|EE-13535-86 P1|Choristoneura fumiferanae|BIN15900
Baryscapus sp.|EE-12482ii-86 P2|Choristoneura fumiferanae|BIN15900
Elasmus atratus|EE-1075-86 P1|Meteorus trachynotus|BIN4404
Elasmus atratus|EE-24-90 MP|Apanteles petrovae|BIN4404
Elasmus atratus|EE-119-88 P2|Apanteles sp.|BIN4404
Elasmus atratus|EE-1044-86 P1|Meteorus trachynotus|BIN4404
Elasmus atratus|EE-1305-88 P3|Meteorus trachynotus|BIN4404
Elasmus atratus|EE-149-88 P3|Apanteles fumiferanae|BIN4404
Elasmus atratus|EE-1078-88 P3|Meteorus trachynotus|BIN4404
Elasmus atratus|EE-13ii-89 MP|Dolichogenidea absona|BIN4404
Elasmus atratus|EE-182iii-87 P1|Apanteles fumiferanae|BIN4404
Elasmus atratus|EE-182ii-87 P1|Apanteles fumiferanae|BIN4404
Elasmus atratus|EE-182i-87 P1|Apanteles fumiferanae|BIN4404
Elasmus atratus|EE-751ii-86 P2|Meteorus trachynotus|BIN4404
Elasmus atratus|EE-777-86 P1|Meteorus trachynotus|BIN4404
Elasmus atratus|EE-718ii-86 P1|Meteorus trachynotus|BIN4404
Elasmus atratus|EE-399-88 P3|Apanteles fumiferanae|BIN4404
Elasmus atratus|EE-1037-86 P2|Meteorus trachynotus|BIN4404
Elasmus atratus|EE-836iii-86 P2|Meteorus trachynotus|BIN4404

Elasmus atratus|EE-399-88 P3|Apanteles fumiferanae|BIN4404
Elasmus atratus|EE-1037-86 P2|Meteorus trachynotus|BIN4404
Elasmus atratus|EE-836iii-86 P2|Meteorus trachynotus|BIN4404
Elasmus atratus|EE-54-89 MP|Dolichogenidea absona|BIN4404
Elasmus atratus|EE-31-90 MP|Dolichogenidea absona|BIN4404
Elasmus atratus|EE-836ii-86 P2|Meteorus trachynotus|BIN4404
Elasmus atratus|EE-836i-86 P2|Meteorus trachynotus|
Baryscapus coerulescens|EE-27-90 MP|Elasmus sp. Via Dolichogenidea absona|BIN4404
Baryscapus coerulescens|EE-26-91 MP|Elasmus sp. Via Dolichogenidea absona|BIN4404
Elasmus atratus|EE-27-91 MP|Apanteles morrisi|
Elasmus atratus|EE-33-90 MP|Apanteles morrisi|BIN4404
Elasmus atratus|EE-241-88 P3|Apanteles fumiferanae|BIN4404
Elasmus atratus|EE-13i-89 MP|Dolichogenidea absona|BIN4404
Elasmus atratus|EE-1153-88 P3|Meteorus trachynotus|BIN4404
Elasmus atratus|EE-14ii-90 MP|Dolichogenidea absona|BIN4404
Elasmus atratus|EE-14i-90 MP|Dolichogenidea absona|BIN4404
Elasmus atratus|EE-648-86 P2|Meteorus trachynotus|BIN4404
Elasmus atratus|EE-856-86 P2|Meteorus trachynotus|BIN4404
Elasmus atratus|EE-1163-88 P3|Meteorus trachynotus|BIN4404
Elasmus atratus|EE-32-87 P1|Apanteles fumiferanae|BIN4404
Elasmus atratus|EE-884ii-86 P1|Meteorus trachynotus|BIN4404
Elasmus atratus|EE-884i-86 P1|Meteorus trachynotus|BIN4404
Elasmus atratus|EE-718i-86 P1|Meteorus trachynotus|BIN4404
Elasmus atratus|EE-862i-86 P1|Meteorus trachynotus|BIN4404
Elasmus atratus|EE-111i-86 P2|Apanteles fumiferanae|BIN4404
Elasmus atratus|EE-91-88 P3|Meteorus trachynotus|BIN4404
Elasmus atratus|EE-1246-88 P3|Meteorus trachynotus|BIN4404
Elasmus atratus|EE-3-95 MP|Dolichogenidea absona|BIN4404
Elasmus atratus|EE-862ii-86 P1|Meteorus trachynotus|BIN4404
Elasmus atratus|EE-1073-86 P1|Meteorus trachynotus|BIN4404
Elasmus atratus|EE-134-88 P3|Apanteles fumiferanae|BIN4404
Elasmus atratus|EE-17ii-87 P1|Apanteles fumiferanae|BIN4404
Elasmus atratus|EE-17i-87 P1|Apanteles fumiferanae|BIN4404
Elasmus atratus|EE-237-88 P3|Apanteles fumiferanae|BIN4404
Elasmus atratus|EE-1485-88 P3|Meteorus trachynotus|BIN4404
Elasmus atratus|EE-83-88 P3|Apanteles fumiferanae|BIN4404
Elasmus atratus|EE-40-89 MP|Dolichogenidea absona|BIN4404
Elasmus atratus|EE-156-88 P3|Apanteles fumiferanae|BIN4404
Perilampus sp.|EE-4391-89 P2|Coleotechnites piceaella|BIN14794
Perilampus sp.|EE-3393-91 P3|Coleotechnites piceaella|BIN14794
Perilampus sp.|EE-3413-91 P2|Choristoneura fumiferanae|BIN14793
Perilampus sp.|EE-733-93 MP|Coleotechnites piceaella|BIN14793
Perilampus sp.|EE-1083-93 MP|Coleotechnites piceaella|BIN14793
Perilampus sp.|EE-2064-92 P2|Coleotechnites piceaella|BIN14793
Perilampus sp.|EE-2777-90 P3|Coleotechnites piceaella|BIN14793
Perilampus sp.|EE-3918-91 P3|Coleotechnites piceaella|BIN14793
Perilampus sp.|EE-5219-88 P2|Coleotechnites piceaella|BIN14793
Perilampus sp.|EE-3414-91 P2|Coleotechnites piceaella|BIN14793
Perilampus sp.|EE-4082-91 P3|Coleotechnites piceaella|BIN14793
Perilampus sp.|EE-2210-89 P3|Coleotechnites piceaella|BIN14793
Perilampus sp.|EE-4627-91 P3|Coleotechnites piceaella|BIN14793
Perilampus sp.|EE-100-89 P1|Coleotechnites piceaella|BIN14793
Perilampus sp.|EE-213-91 MP|Coleotechnites piceaella|BIN14793
Perilampus sp.|EE-3631-89 P1|Coleotechnites piceaella|BIN14793
Perilampus sp.|EE-3614-89 P2|Coleotechnites piceaella|BIN14793
Perilampus sp.|EE-3690-91 P2|Coleotechnites piceaella|BIN14793
Perilampus sp.|EE-3700-91 P2|Coleotechnites piceaella|BIN14793
Perilampus sp.|EE-280-91 MP|Acleris variana|BIN14793
Perilampus sp.|EE-2076-92 P2|Coleotechnites piceaella|BIN14793
Perilampus sp.|EE-3703-91 P2|Coleotechnites piceaella|BIN14793
Aprostocetus esurus|EE-12792ii-86R P2|Choristoneura fumiferana|
Aprostocetus sp.|EE-13207-86 P2|Choristoneura fumiferana|BIN98771
Trichogramma sp.|EE-13-2ii-88 P3|Choristoneura fumiferana|
Trichogramma sp.|EE-3-2i-88 P3|Choristoneura fumiferana|BIN40242
Trichogramma sp.|EE-1-2-88 P3|Choristoneura fumiferana|BIN40242
Trichogramma sp.|EE-10-2-88 P3|Choristoneura fumiferana|
Mesopolobus tortricis|EE-12939-86 P1|Choristoneura fumiferanae|BIN11879
Mesopolobus tortricidis|EE-4395ii-90 P3|Choristoneura fumiferanae|BIN11879
Mesopolobus tortricis|EE-4395i-90 P3|Choristoneura fumiferanae|BIN11879
Mesopolobus tortricis|EE-13090-86 P1|Choristoneura fumiferanae|BIN11879
Mesopolobus tortricis|EE-13146-86 P1|Choristoneura fumiferanae|BIN11879
Mesopolobus tortricis|EE-12813i-86 P2|Choristoneura fumiferanae|BIN11879
Mesopolobus tortricis|EE-13584-86 P2|Choristoneura fumiferanae|BIN11879
Mesopolobus tortricis|EE-12297-86 P2|Choristoneura fumiferanae|BIN11879
Mesopolobus tortricis|EE-10110-86 P2|Choristoneura fumiferanae|BIN11879
Mesopolobus tortricis|EE-6573-91 P3|Choristoneura fumiferanae|BIN11879
Mesopolobus tortricis|EE-4933-89 P3|Choristoneura fumiferanae|BIN11879
Mesopolobus tortricis|EE-4253-90 P3|Choristoneura fumiferanae|BIN11879
Baryscapus sp.|EE-13462i-86 P1|Choristoneura fumiferanae|BIN11879
Mesopolobus tortricis|EE-13153-86 P1|Choristoneura fumiferanae|BIN11879
Mesopolobus tortricis|EE-6357-91 P3|Choristoneura fumiferanae|BIN11879
Mesopolobus tortricis|EE-6293-91 P3|Choristoneura fumiferanae|BIN11879
Mesopolobus tortricis|EE-5988-91 P3|Choristoneura fumiferanae|BIN11879
Mesopolobus tortricis|EE-12796i-86 P2|Choristoneura fumiferanae|BIN11879
Mesopolobus tortricis|EE-12791-86 P2|Choristoneura fumiferanae|BIN11879
Mesopolobus tortricis|EE-12482i-86 P2|Choristoneura fumiferanae|BIN11879
Mesopolobus tortricis|EE-12815i-86 P2|Choristoneura fumiferanae|BIN11879
Mesopolobus tortricis|EE-12924i-86 P1|Choristoneura fumiferanae|BIN11879
Mesopolobus tortricis|EE-12893i-86 P1|Choristoneura fumiferanae|BIN11879
Mesopolobus tortricis|EE-6955-88 P3|Choristoneura fumiferanae|BIN11879
Mesopolobus tortricis|EE-6568-91 P3|Choristoneura fumiferanae|BIN11879
Mesopolobus tortricis|EE-12692-86 P1|Choristoneura fumiferanae|BIN11879
Mesopolobus tortricis|EE-12652-86 P2|Choristoneura fumiferanae|BIN11879
Mesopolobus tortricis|EE-13492-86 P2|Choristoneura fumiferana|BIN11879
Mesopolobus tortricis|EE-12489-86 P2|Choristoneura fumiferanae|BIN11879
Pediobius sp.|EE-13308ii-86 P2|Choristoneura fumiferanae|
Conura albifrons|EE-129-84 P1|Apanteles fumiferanae|BIN97180
Bassus binominatus|EE-10804-86 P2|Coleotechnites piceaella|BIN7526
Bassus binominatus|EE-6841-86 P1|Choristoneura fumiferana|BIN7526
Bassus binominatus|EE-11586-86 P1|Coleotechnites piceaella|BIN7526
Bassus binominatus|EE-320-88 P1|Coleotechnites piceaella|BIN7526
Bassus binominatus|EE-3551-86 P2|Coleotechnites blastovora|BIN7526

Bassus binominatus|EE-11586-86 P1|Coleotechnites piceaella|BIN7526
Bassus binominatus|EE-320-88 P1|Coleotechnites piceaella|BIN7526
Bassus binominatus|EE-3551-86 P2|Coleotechnites blastovora|BIN7526
Bassus binominatus|EE-6140-87 P2|Coleotechnites piceaella|BIN7526
Bassus binominatus|EE-6989-87 P2|Coleotechnites piceaella|BIN7526
Bassus binominatus|EE-7651-87 P2|Coleotechnites piceaella|BIN7526
Bassus binominatus|EE-2394-89 P3|Choristoneura fumiferana|BIN7526
Bassus binominatus|EE-3358-89 P3|Coleotechnites piceaella|BIN7526
Bassus binominatus|EE-922-90 P3|Coleotechnites piceaella|BIN7526
Bassus binominatus|EE-3336-91 P3|Coleotechnites piceaella|BIN7526
Bassus binominatus|EE-3728-91 P3|Coleotechnites piceaella|BIN7526
Bassus binominatus|EE-6154-87 P2|Coleotechnites piceaella|BIN7526
Bassus binominatus|EE-3175-86 P2|Choristoneura fumiferana|BIN7526
Bassus binominatus|EE-141-95 MP|Coleotechnites piceaella|BIN7526
Bassus binominatus|EE-21v-88 P2|Coleotechnites piceaella|BIN7526
Bassus binominatus|EE-6722-86 P2|Choristoneura fumiferana|BIN7526
Bassus binominatus|EE-589-93 MP|Coleotechnites piceaella|BIN7526
Bassus binominatus|EE-4623-91 P3|Coleotechnites piceaella|BIN7526
Bassus binominatus|EE-2519-89 P3|Coleotechnites piceaella|BIN7526
Bassus binominatus|EE-2768-88 P3|Coleotechnites piceaella|BIN7526
Bassus binominatus|EE-3490-91 P3|Choristoneura fumiferana|BIN7526
Bassus binominatus|EE-62-88 P2|Coleotechnites piceaella|BIN7526
Bassus binominatus|EE-7382-87 P2|Coleotechnites piceaella|BIN7526
Bassus binominatus|EE-5809-87 P2|Coleotechnites piceaella|BIN7526
Bassus binominatus|EE-4607-87 P2|Coleotechnites piceaella|BIN7526
Bassus binominatus|EE-4497-86 P2|Choristoneura fumiferana|BIN7526
Bassus binominatus|EE-519-86 P2|Choristoneura fumiferana|BIN7526
Bassus binominatus|EE-331-88 P1|Coleotechnites piceaella|BIN7526
Bassus binominatus|EE-7061-87 P1|Coleotechnites piceaella|BIN7526
Bassus binominatus|EE-2728-87 P1|Coleotechnites piceaella|BIN7526
Bassus binominatus|EE-1426-87 P1|Coleotechnites piceaella|BIN7526
Bassus binominatus|EE-8050-86 P1|Coleotechnites piceaella|BIN7526
Bassus binominatus|EE-695-86 P1|Choristoneura fumiferana|BIN7526
Bassus binominatus|EE-2744-88 P3|Coleotechnites piceaella|BIN7526
Bassus binominatus|EE-217-88 P1|Coleotechnites piceaella|BIN7526
Bassus binominatus|EE-6-92 MP|Coleotechnites piceaella|BIN7526
Bassus binominatus|EE-4950-87 P1|Coleotechnites piceaella|BIN7526
Bassus binominatus|EE-3099-86 P2|Coleotechnites blastovora|BIN7526
Bassus binominatus|EE-1882-89 P3|Coleotechnites piceaella|BIN7526
Bassus binominatus|EE-3065-91 P3|Coleotechnites piceaella|BIN7526
Bassus binominatus|EE-6450-85 P1|Coleotechnites piceaella|BIN7526
Bassus binominatus|EE-327-94 MP|Coleotechnites piceaella|BIN7526
Bassus binominatus|EE-588-89 P2|Epinota radicana|BIN7526
Bassus binominatus|EE-3047-90 P2|Choristoneura fumiferana|BIN7526
Bassus binominatus|EE-1103-93 MP|Coleotechnites piceaella|
Bassus binominatus|EE-143-95 MP|Coleotechnites piceaella|BIN7526
Bassus dimidiator|EE-339-88 P2|Coleotechnites piceaella|BIN10556
Bassus dimidiator|EE-5668-86 P2|Coleotechnites piceaella|BIN10556
Bassus dimidiator|EE-3726-86 P2|Coleotechnites piceaella|BIN10556
Bassus dimidiator|EE-3620-89 P1|Coleotechnites piceaella|BIN10556
Bassus dimidiator|EE-1047-86 P1|Coleotechnites piceaella|BIN10556
Bassus dimidiator|EE-3999-85 P1|Choristoneura fumiferana|BIN10556
Bassus dimidiator|EE-4458-88 P2|Coleotechnites piceaella|BIN10556
Bassus dimidiator|EE-2236-89 P2|Coleotechnites piceaella|BIN10556
Bassus dimidiator|EE-2249-89 P2|Coleotechnites piceaella|BIN10556
Bassus dimidiator|EE-5231-88 P2|Coleotechnites piceaella|BIN10556
Bassus dimidiator|EE-2823-89 P2|Coleotechnites piceaella|BIN10556
Bassus dimidiator|EE-P359-89 MP|Coleotechnites piceaella|BIN10556
Charmon extensor|EE-1808-90 P2|Coleotechnites piceaella|BIN10556
Charmon extensor|EE-196-95 MP|Acleris variana|
Charmon extensor|EE-414-89 MP|Choristoneura fumiferana|
Charmon extensor|EE-162-95 MP|Acleris variana|BIN7527
Charmon extensor|EE-3078-90 P2|Acleris variana|BIN7527
Charmon extensor|EE-295-90 MP|Choristoneura fumiferana|BIN7527
Charmon extensor|EE-P195-90 MP|Choristoneura fumiferana|BIN7527
Charmon extensor|EE-311-90 MP|Choristoneura fumiferana|BIN7527
Charmon extensor|EE-276-90 MP|Choristoneura fumiferana|BIN7527
Charmon extensor|EE-P174-90 MP|Choristoneura fumiferana|BIN7527
Charmon extensor|EE-P130-90 MP|Choristoneura fumiferana|BIN7527
Charmon extensor|EE-P285-90 MP|Choristoneura fumiferana|BIN7527
Charmon extensor|EE-P293-90 MP|Choristoneura fumiferana|BIN7527
Charmon extensor|EE-57-91 MP|Choristoneura fumiferana|BIN7527
Charmon extensor|EE-62-91 MP|Choristoneura fumiferana|BIN7527
Charmon extensor|EE-88-95 MP|Acleris variana|BIN7527
Charmon extensor|EE-206-95 MP|Acleris variana|BIN7527
Bassus binominatus|EE-166-95 MP|Acleris variana|BIN7527
Charmon extensor|EE-3372-90 P2|Acleris variana|BIN7527
Charmon extensor|EE-211-95 MP|Acleris variana|BIN7527
Charmon extensor|EE-4157-88 P3|Coleotechnites piceaella|BIN10555
Charmon extensor|EE-3919-91 P3|Coleotechnites piceaella|BIN10555
Charmon extensor|EE-1411-91 P3|Coleotechnites piceaella|BIN10555
Charmon extensor|EE-6233-86 P1|Coleotechnites piceaella|BIN10555
Charmon extensor|EE-5365-87 P2|Coleotechnites piceaella|BIN10555
Charmon extensor|EE-4994-88 P2|Coleotechnites piceaella|BIN10555
Charmon extensor|EE-5949-88 P2|Coleotechnites piceaella|BIN10555
Charmon extensor|EE-3484-90 P3|Coleotechnites piceaella|BIN10555
Charmon extensor|EE-2410-90 P3|Coleotechnites piceaella|BIN10555
Charmon extensor|EE-4562-87 P2|Coleotechnites piceaella|BIN10555
Charmon extensor|EE-4910-91 P3|Choristoneura fumiferana|BIN10555
Charmon extensor|EE-5687-88 P2|Coleotechnites piceaella|BIN10555
Charmon extensor|EE-2951-91 P3|Coleotechnites piceaella|BIN10555
Charmon sp.|EE-732-93R P3|Epinota radicana|BIN10555
Apanteles morrisi|EE-2095-89 P3|Choristoneura rosaceana|BIN6373
Apanteles morrisi|EE-1556-87 P1|Choristoneura rosaceana|BIN6373
Apanteles morrisi|EE-952-86 P1|Choristoneura fumiferana|BIN6373
Apanteles morrisi|EE-171-89 P2|Choristoneura fumiferana|BIN6373
Apanteles morrisi|EE-3114-92 P3|Choristoneura fumiferana|BIN6373
Apanteles morrisi|EE-246-90 MP|Choristoneura fumiferana|BIN6373
Apanteles morrisi|EE-1279-86 P1|Choristoneura fumiferana|BIN6373
Apanteles morrisi|EE-1302-89 P2|Choristoneura rosaceana|BIN6373
Apanteles morrisi|EE-1603-90 P3|Acleris variana|BIN6373
Apanteles morrisi|EE-1167-90 P3|Choristoneura rosaceana|BIN6373
Apanteles fumiferanae|EE-1087-90 P3|Choristoneura rosaceana|BIN6373

Apanteles morrisi|EE-[illegible] P2|Choristoneura rosaceana|BIN6373
Apanteles morrisi|EE-1603-90 P3|Acleris variana|BIN6373
Apanteles morrisi|EE-1167-90 P3|Choristoneura rosaceana|BIN6373
Apanteles fumiferanae|EE-1087-90 P3|Choristoneura rosaceana|BIN6373
Apanteles fumiferanae|EE-1595-90 P3|Choristoneura rosaceana|BIN6373
Apanteles morrisi|EE-1324-90 P3|Choristoneura fumiferana|BIN6373
Apanteles morrisi|EE-1354-90 P3|Choristoneura rosaceana|BIN6373
Apanteles morrisi|EE-1434-90 P3|Choristoneura rosaceana|BIN6373
Apanteles morrisi|EE-474-89 P3|Choristoneura rosaceana|BIN6373
Apanteles morrisi|EE-1283-89 P3|Choristoneura rosaceana|BIN6373
Apanteles morrisi|EE-1284-89 P3|Choristoneura rosaceana|BIN6373
Apanteles morrisi|EE-1921-89 P3|Choristoneura rosaceana|BIN6373
Apanteles morrisi|EE-7-88 P2|Choristoneura rosaceana|BIN6373
Apanteles morrisi|EE-2527-87 P1|Choristoneura rosaceana|BIN6373
Apanteles morrisi|EE-4048-89 P1|Choristoneura fumiferana|BIN6373
Apanteles morrisi|EE-7511-86 P1|Choristoneura fumiferana|BIN6373
Apanteles morrisi|EE-8351-85 P1|Choristoneura fumiferana|BIN6373
Apanteles morrisi|EE-322-85 P1|Choristoneura fumiferana|BIN6373
Apanteles morrisi|EE-P258-90 MP|Choristoneura fumiferana|BIN6373
Apanteles morrisi|EE-P338-90 MP|Choristoneura fumiferana|BIN6373
Apanteles morrisi|EE-P232-90 MP|Choristoneura fumiferana|BIN6373
Apanteles morrisi|EE-P342-90 MP|Choristoneura fumiferana|BIN6373
Apanteles morrisi|EE-P887-88 MP|Choristoneura fumiferana|BIN6373
Apanteles morrisi|EE-268-86 P2|Choristoneura rosaceana|BIN6373
Apanteles morrisi|EE-8487-86 P2|Choristoneura fumiferana|BIN6373
Apanteles morrisi|EE-1342-87 P1|Choristoneura rosaceana|BIN6373
Apanteles morrisi|EE-6-87 P2|Choristoneura rosaceana|BIN6373
Apanteles morrisi|EE-2-87 P2|Choristoneura rosaceana|BIN6373
Apanteles morrisi|EE-545-87 P2|Choristoneura fumiferana|BIN6373
Apanteles morrisi|EE-1618-87 P1|Choristoneura rosaceana|BIN6373
Apanteles morrisi|EE-43-85 P1|Choristoneura rosaceana|BIN6373
Apanteles morrisi|EE-5282-86 P1|Choristoneura fumiferana|BIN6373
Apanteles morrisi|EE-7975-85 P1|Choristoneura fumiferana|BIN6373
Apanteles morrisi|EE-8014-85 P1|Choristoneura fumiferana|BIN6373
Apanteles morrisi|EE-1364-87 P1|Choristoneura rosaceana|BIN6373
Apanteles morrisi|EE-2658-92 P3|Choristoneura fumiferana|BIN6373
Apanteles morrisi|EE-155-89 P2|Choristoneura fumiferana|BIN6373
Apanteles morrisi|EE-419-88 P2|Choristoneura fumiferana|BIN6373
Apanteles morrisi|EE-384-88 P2|Choristoneura fumiferana|BIN6373
Apanteles morrisi|EE-1343-87 P1|Choristoneura rosaceana|BIN6373
Apanteles morrisi|EE-355-88 P2|Choristoneura fumiferana|BIN6373
Apanteles morrisi|EE-448-88 P2|Choristoneura fumiferana|BIN6373
Apanteles morrisi|EE-393-88 P2|Choristoneura fumiferana|BIN6373
Apanteles morrisi|EE-278-88 P2|Choristoneura rosaceana|BIN6373
Apanteles morrisi|EE-P302-90 MP|Choristoneura fumiferana|BIN6373
Apanteles morrisi|EE-174-89 P2|Choristoneura fumiferana|BIN6373
Apanteles morrisi|EE-5977-85 P1|Choristoneura fumiferana|BIN6373
Apanteles morrisi|EE-P219-90 MP|Choristoneura fumiferana|BIN6373
Apanteles morrisi|EE-P391-90 MP|Choristoneura fumiferana|BIN6373
Apanteles morrisi|EE-P283-90 MP|Choristoneura fumiferana|BIN6375
Apanteles milleri|EE-3121-86 P1|Choristoneura fumiferana|BIN35420
Apanteles milleri|EE-6886-86 P1|Coleotechnites piceaella|BIN35420
Apanteles milleri|EE-5009-86 P2|Coleotechnites piceaella|BIN35420
Apanteles milleri|EE-5444-85 P1|Coleotechnites piceaella|BIN35420
Apanteles milleri|EE-4789-89 P2|Choristoneura fumiferana|BIN35420
Apanteles fumiferanae|EE-4576-88 P3|Choristoneura fumiferana|BIN8400
Apanteles fumiferanae|EE-4902-88 P3|Choristoneura fumiferana|BIN8400
Apanteles fumiferanae|EE-2870-88 P3|Choristoneura fumiferana|BIN8400
Apanteles fumiferanae|EE-341-85 P1|Choristoneura fumiferana|BIN8400
Apanteles fumiferanae|EE-2320-88 P3|Choristoneura fumiferana|BIN8400
Apanteles fumiferanae|EE-2593-88 P3|Choristoneura fumiferana|BIN8400
Apanteles fumiferanae|EE-1482-88 P3|Choristoneura fumiferana|BIN8400
Apanteles fumiferanae|EE-1520-88 P3|Choristoneura fumiferana|BIN8400
Apanteles fumiferanae|EE-13282-86 P2|Coleotechnites piceaella|BIN8400
Apanteles fumiferanae|EE-718-88 P3|Choristoneura fumiferana|BIN8400
Apanteles fumiferanae|EE-10375-86 P2|Choristoneura fumiferana|BIN8400
Apanteles fumiferanae|EE-8861-86 P2|Choristoneura fumiferana|BIN8400
Apanteles fumiferanae|EE-3934-86 P2|Choristoneura fumiferana|BIN8400
Apanteles fumiferanae|EE-94-89 P2|Choristoneura fumiferana|BIN8400
Apanteles fumiferanae|EE-5654-86 P2|Choristoneura fumiferana|BIN8400
Apanteles fumiferanae|EE-5035-86 P2|Choristoneura fumiferana|BIN8400
Apanteles fumiferanae|EE-4695-86 P2|Choristoneura fumiferana|BIN8400
Apanteles fumiferanae|EE-4684-85 P1|Coleotechnites piceaella|BIN8400
Apanteles fumiferanae|EE-8908-85 P1|Choristoneura fumiferana|BIN8400
Apanteles fumiferanae|EE-1099-85 P1|Choristoneura fumiferana|BIN8400
Apanteles fumiferanae|EE-1095-85 P1|Choristoneura fumiferana|BIN8400
Apanteles fumiferanae|EE-1036-85 P1|Choristoneura fumiferana|BIN8400
Apanteles fumiferanae|EE-415-85 P1|Choristoneura fumiferana|BIN8400
Apanteles fumiferanae|EE-132-85 P1|Choristoneura fumiferana|BIN8400
Apanteles fumiferanae|EE-95-85 P1|Choristoneura fumiferana|BIN8400
Apanteles fumiferanae|EE-4415-86 P2|Choristoneura fumiferana|BIN8400
Apanteles fumiferanae|EE-8-85 P1|Choristoneura fumiferana|BIN8400
Apanteles fumiferanae|EE-4442-86 P2|Choristoneura fumiferana|BIN8400
Apanteles fumiferanae|EE-467-85 P1|Choristoneura fumiferana|BIN8400
Apanteles fumiferanae|EE-4632-86 P2|Choristoneura fumiferana|BIN8400
Apanteles fumiferanae|EE-999-88 P3|Choristoneura fumiferana|BIN8400
Apanteles fumiferanae|EE-1063-88 P3|Choristoneura fumiferana|BIN8400
Apanteles fumiferanae|EE-2606-92 P3|Coleotechnites piceaella|BIN8400
Apanteles fumiferanae|EE-PL10C MP|Choristoneura fumiferana|BIN8400
Apanteles fumiferanae|EE-50-88 MP|Choristoneura fumiferana|BIN8400
Apanteles fumiferanae|EE-416-88 MP|Choristoneura fumiferana|BIN8400
Apanteles fumiferanae|EE-963-88 MP|Choristoneura fumiferana|BIN8400
Bassus dimidiator|EE-4909-88 P2|Coleotechnites piceaella|BIN8400
Dolichogenidea absona|EE-297-88 P2|Epinota radicana|BIN8401
Dolichogenidea absona|EE-3056-90 P2|Choristoneura fumiferana|BIN8401
Dolichogenidea absona|EE-3746-88 P1|Choristoneura fumiferana|BIN8401
Dolichogenidea absona|EE-3731-88 P1|Choristoneura fumiferana|BIN8401
Apanteles fumiferanae|EE-4397-88 P2|Acleris variana|BIN8401
Dolichogenidea absona|EE-343-88 P2|Choristoneura fumiferana|BIN8401
Dolichogenidea absona|EE-P85-90 MP|Choristoneura fumiferana|BIN8401
Dolichogenidea absona|EE-2842-89 P1|Choristoneura fumiferana|BIN8401
Apanteles petrovae|EE-P393-90 MP|Choristoneura fumiferana|BIN8401
Dolichogenidea absona|EE-1101-93 MP|Coleotechnites piceaella|BIN8401

Dolichogenidea absona|EE-2842-89 P1|Choristoneura fumiferana|BIN8401
Apanteles petrovae|EE-P393-90 MP|Choristoneura fumiferana|BIN8401
Dolichogenidea absona|EE-1101-93 MP|Coleotechnites piceaella|BIN8401
Dolichogenidea absona|EE-299-90 MP|Coleotechnites piceaella|BIN8401
Dolichogenidea absona|EE-3075-90 P2|Coleotechnites piceaella|BIN8401
Dolichogenidea absona|EE-14-89 P2|Choristoneura fumiferana|BIN8401
Dolichogenidea absona|EE-2549-90 P2|Choristoneura fumiferana|BIN8401
Dolichogenidea absona|EE-2837-89 P1|Acleris variana|BIN8401
Dolichogenidea absona|EE-2274-89 P1|Choristoneura fumiferana|BIN8401
Dolichogenidea absona|EE-3755-88 P1|Choristoneura fumiferana|BIN8401
Dolichogenidea absona|EE-28-89 P2|Choristoneura fumiferana|BIN8401
Dolichogenidea absona|EE-170-88 P2|Choristoneura fumiferana|BIN8401
Dolichogenidea absona|EE-P84-90 MP|Choristoneura fumiferana|BIN8401
Dolichogenidea absona|EE-P6-90 MP|Choristoneura fumiferana|BIN8401
Dolichogenidea absona|EE-P82-90 MP|Choristoneura fumiferana|BIN8401
Dolichogenidea absona|EE-P57-90 MP|Choristoneura fumiferana|BIN8401
Dolichogenidea absona|EE-489-88 P2|Choristoneura fumiferana|BIN8401
Dolichogenidea absona|EE-21-88 P2|Choristoneura fumiferana|BIN8401
Dolichogenidea absona|EE-5244-88 P2|Coleotechnites piceaella|BIN8401
Dolichogenidea absona|EE-P10-90 MP|Choristoneura fumiferana|BIN8401
Dolichogenidea absona|EE-P20-90 MP|Choristoneura fumiferana|BIN8401
Dolichogenidea absona|EE-P43-90 MP|Choristoneura fumiferana|BIN8401
Dolichogenidea absona|EE-2273-89 P1|Choristoneura fumiferana|BIN8401
Apanteles petrovae|EE-P162-89 MP|Choristoneura fumiferana|
Dolichogenidea absona|EE-2806-90 P2|Choristoneura fumiferana|BIN8401
Dolichogenidea absona|EE-P48-90 MP|Choristoneura fumiferana|BIN8401
Dolichogenidea absona|EE-P94-90 MP|Choristoneura fumiferana|BIN8401
Dolichogenidea renaulti|EE-603-93 P3|Epinota radicana|BIN4312
Dolichogenidea renaulti|EE-2808-90 P2|Coleotechnites piceaella|BIN6372
Dolichogenidea renaulti|EE-3044-90 P2|Coleotechnites piceaella|BIN6372
Dolichogenidea renaulti|EE-4954-88 P2|Acleris variana|BIN5833
Dolichogenidea renaulti|EE-4987-88 P2|Acleris variana|BIN5833
Dolichogenidea renaulti|EE-5195-88 P2|Acleris variana|BIN5833
Dolichogenidea renaulti|EE-2788-90 P2|Acleris variana|BIN5833
Dolichogenidea renaulti|EE-543-93 P3|Acleris variana|BIN5833
Dolichogenidea renaulti|EE-1115-93 MP|Acleris variana|BIN5833
Dolichogenidea renaulti|EE-231-94 MP|Acleris variana|BIN5833
Dolichogenidea renaulti|EE-430-93 P3|Acleris variana|BIN5833
Dolichogenidea renaulti|EE-3070-90 P2|Acleris variana|BIN5833
Dolichogenidea renaulti|EE-4953-88 P2|Acleris variana|BIN5833
Dolichogenidea renaulti|EE-173-95 MP|Acleris variana|BIN5833
Apanteles petrovae|EE-16-91 MP|Choristoneura fumiferana|BIN6374
Apanteles petrovae|EE-21-91 MP|Choristoneura fumiferana|BIN6374
Apanteles petrovae|EE-42-91 MP|Choristoneura fumiferana|BIN6374
Apanteles petrovae|EE-41-91 MP|Choristoneura fumiferana|BIN6374
Apanteles petrovae|EE-5068-88 P2|Choristoneura fumiferana|BIN6374
Apanteles petrovae|EE-7197-86 P2|Choristoneura fumiferana|BIN6374
Apanteles petrovae|EE-3013-87 P1|Choristoneura fumiferana|BIN6374
Apanteles petrovae|EE-12737-85 P1|Choristoneura fumiferana|BIN6374
Apanteles petrovae|EE-249-89 P2|Choristoneura fumiferana|BIN6374
Apanteles morrisi|EE-P270-88 P2|Choristoneura fumiferana|BIN6374
Apanteles petrovae|EE-251-89 P2|Choristoneura fumiferana|BIN6374
Apanteles petrovae|EE-260-89 P2|Choristoneura fumiferana|BIN6374
Apanteles petrovae|EE-163-91 MP|Choristoneura fumiferana|BIN6374
Apanteles petrovae|EE-311-91 MP|Choristoneura fumiferana|BIN6374
Apanteles petrovae|EE-323-91 MP|Choristoneura fumiferana|BIN6374
Microgaster sp.|EE-319-88 MP|Choristoneura fumiferana|BIN7886
Microgaster sp.|EE-993-P1-93 P2|Choristoneura fumiferana|BIN7886
Microgaster sp.|EE-5439-88 P2|Acleris variana|BIN7887
Orgilus sp.|EE-4569-87 P2|Coleotechnites piceaella|BIN7696
Orgilus sp. 1-MJS|EE-12-89R P2|Coleotechnites piceaella|BIN7696
Orgilus sp.|EE-3931-89 P3|Coleotechnites piceaella|BIN7695
Orgilus sp.|EE-308-88 P2|Coleotechnites piceaella|BIN7695
Orgilus sp.|EE-252-87 P2|Coleotechnites piceaella|BIN7695
Orgilus sp.|EE-292-88 P1|Coleotechnites piceaella|BIN7695
Orgilus sp.|EE-286-88 P1|Coleotechnites piceaella|BIN7695
Orgilus sp.|EE-3039-90 P3|Coleotechnites piceaella|BIN7695
Orgilus sp.|EE-2665-90 P3|Coleotechnites piceaella|BIN7695
Orgilus sp.|EE-87-90 P3|Coleotechnites piceaella|BIN7695
Bassus dimidiator|EE-2920-90 P3|Coleotechnites piceaella|BIN7695
Orgilus sp.|EE-143-87 P2|Coleotechnites piceaella|
Orgilus sp.|EE-633-93 P3|Coleotechnites piceaella|BIN7695
Orgilus sp.|EE-4404-88 P2|Coleotechnites piceaella|BIN7695
Orgilus sp.|EE-12440-85 P1|Coleotechnites piceaella|BIN7695
Orgilus sp.|EE-4957-86 P2|Coleotechnites piceaella|BIN7695
Orgilus sp.|EE-6626-87 P2|Coleotechnites piceaella|BIN7695
Orgilus sp.|EE-340-88 P2|Coleotechnites piceaella|BIN7695
Orgilus sp.|EE-2579-89 P3|Coleotechnites piceaella|BIN7695
Orgilus sp.|EE-3930-89 P3|Coleotechnites piceaella|BIN7695
Orgilus sp.|EE-966-93 MP|Coleotechnites piceaella|BIN7695
Orgilus sp.|EE-135-95 MP|Coleotechnites piceaella|BIN7695
Orgilus sp.|EE-864-93 MP|Coleotechnites piceaella|BIN7695
Orgilus sp.|EE-1025-93 MP|Coleotechnites piceaella|BIN7695
Orgilus sp.|EE-3-92 MP|Coleotechnites piceaella|BIN7695
Orgilus sp.|EE-191-92 MP|Coleotechnites piceaella|BIN7695
Orgilus sp.|EE-2698-89 P3|Coleotechnites piceaella|BIN7695
Orgilus sp.|EE-4225-89 P3|Coleotechnites piceaella|BIN7695
Orgilus sp.|EE-3536-86 P2|Coleotechnites blastovora|BIN7695
Orgilus sp.|EE-6911-87 P2|Coleotechnites piceaella|BIN7695
Orgilus sp.|EE-5026-87 P2|Coleotechnites piceaella|BIN7695
Orgilus sp.|EE-298-88 P1|Coleotechnites piceaella|BIN7695
Orgilus sp.|EE-7565-87 P1|Coleotechnites piceaella|BIN7695
Orgilus sp.|EE-7062-87 P1|Coleotechnites piceaella|BIN7695
Orgilus sp.|EE-6470-87 P1|Coleotechnites piceaella|BIN7695
Orgilus sp.|EE-7301-86 P1|Coleotechnites piceaella|BIN7695
Orgilus sp.|EE-14292-85 P1|Coleotechnites piceaella|BIN7695
Orgilus sp.|EE-9353-85 P1|Coleotechnites piceaella|BIN7695
Orgilus sp.|EE-1694-90 P3|Coleotechnites piceaella|BIN7695
Orgilus sp.|EE-1687-90 P3|Coleotechnites piceaella|BIN7695
Orgilus sp.|EE-3363-89 P2|Acleris variana|BIN7695
Orgilus sp.|EE-4098-91 P3|Choristoneura fumiferana|BIN7695
Orgilus sp.|EE-404-93 P3|Coleotechnites piceaella|BIN7695
Orgilus sp.|EE-81-88 P2|Coleotechnites piceaella|

Orgilus sp.|EE-[illegible] P2|Acleris variana|BIN7695
Orgilus sp.|EE-4098-91 P3|Choristoneura fumiferana|BIN7695
Orgilus sp.|EE-404-93 P3|Coleotechnites piceaella|BIN7695
Orgilus sp.|EE-81-88 P2|Coleotechnites piceaella|
Orgilus sp.|EE-197-93 P3|Acleris variana|BIN7695
Orgilus sp.|EE-318-93 P3|Coleotechnites piceaella|BIN7695
Orgilus sp.|EE-513-93 P3|Coleotechnites piceaella|BIN7695
Orgilus sp. 2-MJS|EE-28-88R P2|Coleotechnites piceaella|BIN7695
Ascogaster provancheri|EE-174-95 MP|Acleris variana|BIN16615
Ascogaster provancheri|EE-736-93 P3|Epinota radicana|
Ascogaster provancheri|EE-417-93 P3|Acleris variana|BIN16615
Ascogaster provancheri|EE-163-95 MP|Acleris variana|BIN16615
Ascogaster provancheri|EE-644-93R P3|Choristoneura fumiferana|BIN16615
Chelonus sp.|EE-3485-90 P3|Acleris variana|BIN16615
Chelonus sp.|EE-679-90 P3|Coleotechnites piceaella|BIN16616
Apanteles fumiferanae|EE-3604-89 P3|Coleotechnites piceaella|BIN8402
Chelonus sp.|EE-12258-85 P1|Coleotechnites piceaella|BIN8402
Chelonus sp.|EE-3155-91 P3|Choristoneura fumiferana|BIN8402
Chelonus sp.|EE-2194-89 P3|Coleotechnites piceaella|BIN8402
Chelonus sp.|EE-419-90 P3|Coleotechnites piceaella|BIN8402
Chelonus sp.|EE-1689-90 P3|Coleotechnites piceaella|BIN8402
Chelonus sp.|EE-1725-90 P3|Coleotechnites piceaella|BIN8402
Chelonus sp.|EE-2075-92 P2|Coleotechnites piceaella|BIN8402
Chelonus sp.|EE-3201-87 P2|Coleotechnites piceaella|BIN8402
Chelonus sp.|EE-620-90 P3|Coleotechnites piceaella|BIN8402
Chelonus sp.|EE-1668-90 P3|Coleotechnites piceaella|BIN8402
Chelonus sp.|EE-648-90 P3|Coleotechnites piceaella|BIN8402
Chelonus sp.|EE-77-90 P3|Coleotechnites piceaella|BIN8402
Chelonus sp.|EE-1081-92 P3|Choristoneura fumiferana|BIN8402
Chelonus sp.|EE-1290-87 P1|Coleotechnites piceaella|BIN8402
Chelonus sp.|EE-946-90 P3|Coleotechnites piceaella|BIN8402
Chelonus sp.|EE-260-88 P1|Coleotechnites piceaella|
Chelonus sp.|EE-381-93 P3|Coleotechnites piceaella|BIN8402
Macrocentrus sp.|EE-992-89R P2|Choristoneura fumiferana|BIN48368
Macrocentrus sp.|EE-1853-89R P2|Choristoneura rosaceana|BIN48368
Macrocentrus sp.|EE-1898-89R P2|Choristoneura rosaceana|BIN48369
Meteorus trachynotus|EE-17553-85 P1|Choristoneura fumiferana|BIN9219
Meteorus trachynotus|EE-4350-89 P3|Choristoneura fumiferana|BIN9219
Meteorus trachynotus|EE-10473-86 P2|Choristoneura fumiferana|BIN9219
Meteorus trachynotus|EE-8164-86 P2|Choristoneura fumiferana|BIN9219
Meteorus trachynotus|EE-4249-89 P3|Choristoneura fumiferana|BIN9219
Meteorus trachynotus|EE-4484-89 P3|Choristoneura fumiferana|BIN9219
Meteorus trachynotus|EE-1716-90 P3|Choristoneura rosaceana|
Meteorus trachynotus|EE-378-90 MP|Choristoneura fumiferana|BIN9219
Meteorus trachynotus|EE-375-90 MP|Choristoneura fumiferana|BIN9219
Meteorus trachynotus|EE-19-90 P3|Choristoneura rosaceana|BIN9219
Meteorus trachynotus|EE-1493-88 P3|Choristoneura rosaceana|BIN9219
Meteorus trachynotus|EE-4162-89 P3|Epinota radicana|BIN9219
Meteorus trachynotus|EE-4510-89 P3|Choristoneura fumiferana|BIN9219
Meteorus trachynotus|EE-4078-89 P3|Choristoneura fumiferana|BIN9219
Meteorus trachynotus|EE-6365-88 P3|Choristoneura fumiferana|BIN9219
Meteorus trachynotus|EE-6243-88 P3|Choristoneura fumiferana|BIN9219
Meteorus trachynotus|EE-6030-88 P3|Choristoneura fumiferana|BIN9219
Meteorus trachynotus|EE-5994-88 P3|Choristonuera fumiferana|BIN9219
Meteorus trachynotus|EE-P866-88 MP|Choristoneura fumiferana|BIN9219
Meteorus trachynotus|EE-10769-86 P2|Choristoneura fumiferana|BIN9219
Meteorus trachynotus|EE-10089-86 P2|Choristoneura fumiferana|BIN9219
Meteorus trachynotus|EE-17576-85 P1|Choristoneura fumiferana|BIN9219
Meteorus trachynotus|EE-17502-85 P1|Choristoneura fumiferana|BIN9219
Meteorus trachynotus|EE-17294-85 P1|Choristoneura fumiferana|BIN9219
Meteorus trachynotus|EE-17127-85 P1|Choristoneura fumiferana|BIN9219
Meteorus trachynotus|EE-17124-85 P1|Choristoneura fumiferana|BIN9219
Meteorus trachynotus|EE-16938-85 P1|Choristoneura fumiferana|BIN9219
Meteorus trachynotus|EE-16952-85 P1|Choristoneura fumiferana|BIN9219
Meteorus trachynotus|EE-5589-88 P3|Choristoneura fumiferana|BIN9219
Meteorus trachynotus|EE-12306-86 P2|Choristoneura fumiferana|BIN9219
Meteorus trachynotus|EE-11763-86 P2|Choristoneura fumiferana|BIN9219
Meteorus trachynotus|EE-9381-86 P2|Choristoneura fumiferana|BIN9219
Meteorus trachynotus|EE-8347-85 P1|Choristoneura rosaceana|BIN9219
Meteorus trachynotus|EE-16514-85 P1|Choristoneura fumiferana|BIN9219
Meteorus trachynotus|EE-10750-86 P2|Choristoneura fumiferana|BIN9219
Meteorus trachynotus|EE-11336-86 P2|Choristoneura fumiferana|BIN9219
Meteorus trachynotus|EE-5487-85 P1|Choristoneura rosaceana|BIN9219
Meteorus trachynotus|EE-11348-86 P2|Choristoneura fumiferana|BIN9219
Meteorus trachynotus|EE-17292-85 P1|Choristoneura fumiferana|
Meteorus trachynotus|EE-634-89 P2|Choristoneura fumiferana|BIN9219
Clinocentrus fumiferanae|EE-1119-92 MP|Choristoneura fumiferana|BIN27824
Clinocentrus fumiferanae|EE-534-91 MP|Choristoneura fumiferana|BIN27824
Clinocentrus fumiferanae|EE-479-91 MP|Choristoneura fumiferana|BIN27824
Clinocentrus fumiferanae|EE-559-91 MP|Choristoneura fumiferana|BIN27824
Clinocentrus fumiferanae|EE-204-94 MP|Choristoneura fumiferana|BIN27824
Clinocentrus fumiferanae|EE-383-90 MP|Choristoneura fumiferana|BIN27824
Clinocentrus fumiferanae|EE-370-90 MP|Choristoneura fumiferana|BIN27824
Clinocentrus fumiferanae|EE-395-90 MP|Choristoneura fumiferana|BIN27824
Clinocentrus fumiferanae|EE-405-90 MP|Choristoneura fumiferana|BIN27824
Clinocentrus fumiferanae|EE-275-94 MP|Choristoneura fumiferana|BIN27824
Meteorus sp.|EE-5706-88R P2|Coleotechnites piceaella|
Asobara sp.|EE-853ii-92 P3|Tachinid sp.|
Asobara sp.|EE-375-92 P3|Tachinid sp.|
Asobara sp.|EE-984i-91 P3|Tachinid sp.|BIN40947
Asobara sp.|EE-984ii-91 P3|Tachinid sp.|BIN40947
Asobara sp.|EE-659-92 P3|Tachinid sp.|BIN40947
Asobara sp.|EE-853i-92 P3|Tachinid sp.|
Asobara sp.|EE-573-91 P3|Tachinid sp.|
Asobara sp.|EE-657-91 P3|Tachinid sp.|
Asobara sp.|EE-853iii-92 P3|Tachinid sp.|
Asobara sp.|EE-9-84-A P1|Agria affinis|
Pediobius crassicornis|EE-6549iii-91R P3|Mesopolobus tortricis|
Brachymeria compsilurae|EE-13-1B-84 P2|Smidtia fumiferanae|BIN97973
Phytodietus vulgaris|EE-E44-95 P2|Choristoneura fumiferana|BIN60379
Ichneumonidae|EE-6850-88 P2|Acleris variana|BIN71847
Ichneumonidae|EE-4169-89 P2|Acleris variana|BIN71847
Chelonus sp.|EE-2821-89 R P3|Coleotechnites piceaella|

Ichneumonidae|EE-6850-88 P2|Acleris variana|BIN71847
Ichneumonidae|EE-4169-89 P2|Acleris variana|BIN71847
Chelonus sp.|EE-2821-89 R P3|Coleotechnites piceaella|
Glypta fumiferanae|EE-954-P1-93 MP|Choristoneura fumiferana|BIN5821
Glypta fumiferanae|EE-932-93 MP|Choristoneura fumiferana|BIN5821
Glypta fumiferanae|EE-944-P1-93 MP|Choristoneura fumiferana|BIN5821
Glypta fumiferanae|EE-957-93 MP|Choristoneura fumiferana|BIN5821
Glypta fumiferanae|EE-494-86 P2|Choristoneura fumiferana|BIN5819
Glypta fumiferanae|EE-886-86 P2|Choristoneura fumiferana|BIN5819
Glypta fumiferanae|EE-3283-86 P2|Choristoneura fumiferana|BIN5819
Glypta fumiferanae|EE-2501-85 P1|Choristoneura fumiferana|BIN5819
Glypta fumiferanae|EE-3276-88 P3|Choristoneura fumiferana|BIN5819
Glypta fumiferanae|EE-2032-88 P3|Choristoneura fumiferana|BIN5819
Glypta fumiferanae|EE-1344-88 P3|Choristoneura fumiferana|BIN5819
Glypta fumiferanae|EE-479-88 P3|Choristoneura fumiferana|BIN5819
Glypta fumiferanae|EE-8807-86 P2|Choristoneura fumiferana|BIN5819
Glypta fumiferanae|EE-8525-86 P2|Choristoneura fumiferana|BIN5819
Glypta fumiferanae|EE-5743-86 P2|Choristoneura fumiferana|BIN5819
Glypta fumiferanae|EE-3850-86 P2|Choristoneura fumiferana|BIN5819
Glypta fumiferanae|EE-2108-86 P2|Choristoneura fumiferana|BIN5819
Glypta fumiferanae|EE-1940-86 P2|Choristoneura fumiferana|BIN5819
Glypta fumiferanae|EE-5873-85 P1|Choristoneura fumiferana|BIN5819
Glypta fumiferanae|EE-5049-85 P1|Choristoneura fumiferana|BIN5819
Glypta fumiferanae|EE-5-85 P1|Choristoneura fumiferana|BIN5819
Glypta fumiferanae|EE-3300-85 P1|Choristoneura fumiferana|BIN5819
Glypta fumiferanae|EE-3132-88 P3|Choristoneura fumiferana|BIN5819
Glypta fumiferanae|EE-8033-85 P1|Choristoneura fumiferana|BIN5819
Glypta fumiferanae|EE-740-85 P1|Choristoneura fumiferana|BIN5819
Glypta fumiferanae|EE-8968-86 P2|Choristoneura fumiferana|BIN5819
Glypta fumiferanae|EE-1785-85 P1|Choristoneura fumiferana|BIN5819
Glypta fumiferanae|EE-3900-88 P3|Choristoneura fumiferana|BIN5819
Glypta fumiferanae|EE-4527-88 P3|Choristoneura fumiferana|BIN5819
Glypta fumiferanae|EE-4837-88 P3|Choristoneura fumiferana|BIN5819
Glypta fumiferanae|EE-5353-88 P3|Choristoneura fumiferana|BIN5819
Glypta fumiferanae|EE-6058-88 P3|Choristoneura fumiferana|BIN5819
Glypta sp.|EE-1308- 89 P3|Choristoneura rosaceana|BIN5819
Glypta sp.|EE-1174-89 P3|Choristoneura rosaceana|BIN5819
Glypta sp.|EE-1997-88 P3|Choristoneura rosaceana|BIN5819
Glypta sp.|EE-2000-88 P3|Choristoneura rosaceana|BIN5819
Glypta sp.|EE-2676-88 P3|Choristoneura rosaceana|BIN5819
Glypta sp.|EE-349-89 P3|Choristoneura rosaceana|BIN5819
Glypta sp.|EE-1592-89 P3|Choristoneura rosaceana|BIN5819
Glypta sp.|EE-572-90 P3|Choristoneura rosaceana|BIN5819
Glypta sp.|EE-3577-85 P1|Choristoneura rosaceana|BIN5819
Glypta sp.|EE-5369-85 P1|Choristoneura rosaceana|BIN5819
Glypta fumiferanae|EE-1982-89 P3|Choristoneura rosaceana|BIN5819
Glypta fumiferanae|EE-1966-89 P3|Choristoneura rosaceana|BIN5819
Glypta fumiferanae|EE-1931-89 P3|Choristoneura rosaceana|BIN5819
Glypta fumiferanae|EE-1922-89 P3|Choristoneura rosaceana|BIN5819
Glypta fumiferanae|EE-1471-89 P3|Choristoneura rosaceana|BIN5819
Glypta fumiferanae|EE-1304-89 P3|Choristoneura rosaceana|BIN5819
Glypta fumiferanae|EE-1291-89 P3|Choristoneura rosaceana|BIN5819
Glypta fumiferanae|EE-373-89 P3|Choristoneura rosaceana|BIN5819
Glypta fumiferanae|EE-1554-88 P3|Choristoneura rosaceana|BIN5819
Glypta fumiferanae|EE-1784-90R P2|Choristoneura rosaceana|BIN5819
Glypta fumiferanae|EE-1780-90R P2|Choristoneura rosaceana|BIN5819
Glypta fumiferanae|EE-13938-85 P1|Choristoneura rosaceana|BIN5819
Glypta sp.|EE-3031-86 P2|Choristoneura rosaceana|BIN5819
Glypta fumiferanae|EE-P408-89 MP|Acleris variana|BIN5819
Glypta fumiferanae|EE-1297-89 P3|Choristoneura rosaceana|BIN5819
Glypta sp.|EE-1312-89 P3|Choristoneura rosaceana|BIN5819
Glypta sp.|EE-1030-90 P3|Choristoneura rosaceana|BIN5819
Glypta fumiferanae|EE-3362-89 P2|Acleris variana|BIN5820
Glypta fumiferanae|EE-4649-88 P1|Acleris variana|BIN5820
Glypta fumiferanae|EE-6071-88 P2|Acleris variana|BIN5820
Glypta sp.|EE-3830-89 P2|Acleris variana|
Glypta fumiferanae|EE-4938-88 P2|Acleris variana|BIN5820
Glypta sp.|EE-4051-89 P1|Acleris variana|BIN5820
Glypta fumiferanae|EE-4433-88 P2|Acleris variana|BIN5820
Glypta fumiferanae|EE-5027-88 P2|Acleris variana|BIN5820
Glypta fumiferanae|EE-5399-88 P1|Acleris variana|BIN5820
Glypta sp.|EE-4165-89 P2|Acleris variana|BIN5820
Glypta fumiferanae|EE-3611-89 P2|Acleris variana|BIN5820
Glypta fumiferanae|EE-3386-89 P2|Acleris variana|BIN5820
Glypta fumiferanae|EE-3627-89 P1|Acleris variana|BIN5820
Glypta fumiferanae|EE-3135-89 P1|Acleris variana|BIN5820
Glypta fumiferanae|EE-3639-89 P1|Acleris variana|BIN5820
Glypta fumiferanae|EE-P642-89 MP|Acleris variana|BIN5820
Glypta sp.|EE-3153-89 P1|Acleris variana|BIN5820
Glypta fumiferanae|EE-5204-88 P2|Acleris variana|BIN5820
Glypta fumiferanae|EE-6439-88 P2|Acleris variana|BIN5820
Glypta fumiferanae|EE-3828-89 P2|Acleris variana|BIN5820
Glypta sp.|EE-10701-86 P2|Acleris variana|BIN5820
Glypta sp.|EE-4166-89 P2|Acleris variana|BIN5820
Lissonota sexcincta recurvariae|EE-7488-86 P1|Coleotechnites piceaella|BIN38806
Lissonota sexcincta recurvariae|EE-4730-87 P1|Coleotechnites piceaella|BIN38806
Lissonota sexcincta recurvariae|EE-6434-88 P2|Coleotechnites piceaella|
Lissonota sexcincta recurvariae|EE-2670-90 P3|Coleotechnites piceaella|BIN38805
Lissonota sexcincta recurvariae|EE-1388-90R P3|Coleotechnites piceaella|BIN38805
Lissonota acrobasidis|EE-392-93 P3|Coleotechnites piceaella|BIN38805
Lissonota acrobasidis|EE-3041-91R P3|Choristoneura fumiferana|
Lissonota acrobasidis|EE-27-90 P3|Coleotechnites piceaella|
Lissonota sexcincta recurvariae|EE-2170-90R P3|Coleotechnites piceaella|
Lissonota sexcincta recurvariae|EE-407-93 P3|Coleotechnites piceaella|BIN38805
Stictopisthus sp.|EE-886-92 P3|Meteorus trachynotus|BIN5522
Stictopisthus sp.|EE-1511-89 P3|Glytapanteles sp.|BIN5524
Stictopisthus sp.|EE-107-88 MP|Meteorus trachynotus|BIN5523
Stictopisthus lanceolatus|EE-P369-89 P2|Choristoneura fumiferana via A. morrisi|BIN5523
Stictopisthus lanceolatus|EE-P627-89 P2|Choristoneura fumiferana via Apanteles sp.|BIN5523
Stictopisthus sp.|EE-10-94 MP|Dolichogenidea absona|BIN5523
Stictopisthus sp.|EE-38-89 MP|Dolichogenidea absona|BIN5523
Stictopisthus sp.|EE-240-91 P3|Apanteles fumiferanae|BIN5519
[illegible]

Stictopisthus sp.|EE-10-94 MP|Dolichogenidea absona|BIN5523
Stictopisthus sp.|EE-38-89 MP|Dolichogenidea absona|BIN5523
Stictopisthus sp.|EE-240-91 P3|Apanteles fumiferanae|BIN5519
Stictopisthus sp.|EE-110-91 P3|Apanteles fumiferanae|BIN5519
Stictopisthus sp.|EE-287-91 P3|Apanteles fumiferanae|BIN5519
Stictopisthus sp.|EE-3734-89 P3|Choristoneura fumiferana via Apanteles sp.|BIN5519
Stictopisthus sp.|EE-2534-89 P3|Choristoneura fumiferana via Apanteles sp.|BIN5519
Stictopisthus sp.|EE-538-89 P3|Apanteles fumiferanae|BIN5519
Stictopisthus flaviceps|EE-2451-92R P2|Choristoneura fumiferana via ?|BIN5519
Stictopisthus flaviceps|EE-2441-92R P3|Choristoneura fumiferana via ?|BIN5519
Stictopisthus sp.|EE-58-89 P3|Apanteles fumiferanae|BIN5519
Stictopisthus sp.|EE-99-88 MP|Meteorus trachynotus|BIN5517
Stictopisthus sp.|EE-6496-87 P1|Choristoneura fumiferana via Glypta sp.|BIN5517
Stictopisthus sp.|EE-3827-92 P2|Choristoneura fumiferana via ?|BIN5517
Stictopisthus sp.|EE-105-88 MP|Meteorus trachynotus|BIN5517
Stictopisthus sp.|EE-89-88 MP|Meteorus trachynotus|BIN5517
Stictopisthus sp.|EE-1133-91 P3|Glypta fumiferanae|BIN5517
Stictopisthus sp.|EE-104-88 MP|Meteorus trachynotus|BIN5517
Stictopisthus sp.|EE-690-87 P2|Meteorus trachynotus|BIN5517
Stictopisthus lanceolatus|EE-P891-88 P2|Choristoneura fumiferana via Tranosema|
Stictopisthus sp.|EE-223-87 P2|Meteorus trachynotus|BIN5517
Stictopisthus sp.|EE-167-91 P3|Glypta fumiferanae|BIN5517
Stictopisthus lanceolatus|EE-P796-88 P2|Choristoneura fumiferana via A. morrisi|
Stictopisthus lanceolatus|EE-P829-88 P2|Choristoneura fumiferana via Apanteles sp.|
Stictopisthus lanceolatus|EE-P843-88 P2|Choristoneura fumiferana via A. morrisi|BIN5520
Stictopisthus sp.|EE-5455-88 P2|Choristoneura fumiferana via Apanteles sp.|BIN5520
Stictopisthus sp.|EE-55-89 MP-02|Apanteles morrisi|BIN5520
Stictopisthus sp.|EE-97-88 MP|Apanteles sp.|BIN5525
Stictopisthus sp.|EE-35-88 MP|Apanteles sp.|BIN5521
Stictopisthus sp.|EE-112-88 MP|Apanteles sp.|BIN5521
Stictopisthus lanceolatus|EE-P620-89 P2|Choristoneura fumiferana via A. morrisi|BIN5521
Stictopisthus sp.|EE-51-88 MP|Apanteles sp.|BIN5521
Stictopisthus sp.|EE-39-88 MP|Apanteles sp.|BIN5521
Stictopisthus sp.|EE-37-88 MP|Apanteles sp.|BIN5521
Stictopisthus sp.|EE-586-87 P2|Apanteles fumiferanae|BIN5521
Stictopisthus sp.|EE-82-88 MP|Meteorus trachynotus|BIN5518
Stictopisthus sp.|EE-182-91 P3|Apanteles fumiferanae|BIN5518
Stictopisthus sp.|EE-144-87 P2|Apanteles fumiferanae|BIN5518
Stictopisthus sp.|EE-8-89 MP|Dolichogenidea absona|BIN5518
Stictopisthus sp.|EE-5463-87 P2|Choristoneura fumiferana via Apanteles sp.|BIN5518
Stictopisthus sp.|EE-6089-87 P2|Choristoneura fumiferana via Apanteles sp.|BIN5518
Stictopisthus sp.|EE-6107-87 P2|Choristoneura fumiferana via Apanteles sp.|BIN5518
Stictopisthus sp.|EE-772-93 P2|Acleris variana via Microgaster sp.|BIN5518
Stictopisthus sp.|EE-151-87 P2|Apanteles fumiferanae|BIN5518
Stictopisthus sp.|EE-474-86 P2|Apanteles fumiferanae|BIN5518
Stictopisthus sp.|EE-290-91 P3|Apanteles fumiferanae|BIN5518
Stictopisthus sp.|EE-685-87 P2|Apanteles fumiferanae|BIN5518
Stictopisthus sp.|EE-581-91 P3|Apanteles fumiferanae|BIN5518
Stictopisthus sp.|EE-482-91 P3|Apanteles fumiferanae|BIN5518
Stictopisthus sp.|EE-154-87 P2|Apanteles fumiferanae|BIN5518
Stictopisthus sp.|EE-48-89 MP|Dolichogenidea absona|BIN5518
Stictopisthus sp.|EE-2-94 MP|Dolichogenidea absona|BIN5518
Stictopisthus sp.|EE-21-94 MP|Dolichogenidea absona|BIN5518
Stictopisthus sp.|EE-2-88 P1|Dolichogenidea absona|BIN5518
Stictopisthus sp.|EE-36-88 MP|Apanteles sp.|BIN5518
Stictopisthus sp.|EE-6220-88 P2|Acleris variana via Microgaster sp.|BIN5518
Stictopisthus sp.|EE-3376-90 P2|Coleotechnites piceaella via Orgilus sp.|BIN5518
Stictopisthus sp.|EE-7168-87 P2|Choristoneura fumiferana via Apanteles sp.|BIN5518
Stictopisthus sp.|EE-138-87 P2|Apanteles fumiferanae|BIN5518
Stictopisthus sp.|EE-24-87 P2|Apanteles fumiferanae|BIN5518
Stictopisthus sp.|EE-19-89 MP|Dolichogenidea absona|BIN5518
Stictopisthus sp.|EE-119-87 P2|Apanteles fumiferanae|BIN5518
Stictopisthus sp.|EE-318-87 P2|Apanteles fumiferanae|BIN5518
Stictopisthus sp.|EE-340-84 P1|Apanteles fumiferanae|BIN5518
Stictopisthus sp.|EE-5440-87 P2|Choristoneura fumiferana via Apanteles sp.|BIN5518
Stictopisthus sp.|EE-4771-88 P3|Choristoneura fumiferana via Apanteles sp.|BIN5518
Stictopisthus sp.|EE-668-91 P3|Apanteles fumiferanae|BIN5518
Stictopisthus sp.|EE-469-84 P1|Apanteles fumiferanae|BIN5518
Stictopisthus sp.|EE-33-88 MP|Apanteles sp.|
Stictopisthus lanceolatus|EE-P392-90 P2|Choristoneura fumiferana via A. morrisi|
Stictopisthus lanceolatus|EE-P371-90 P2|Choristoneura fumiferana via Apanteles sp.|BIN5518
Stictopisthus lanceolatus|EE-P802-88 P2|Choristoneura fumiferana via Apanteles sp.|BIN5518
Stictopisthus sp.|EE-188-95 MP|Coleotechnites piceaella|BIN5518
Stictopisthus lanceolatus|EE-P881-88 P2|Choristoneura fumiferana via Apanteles sp.|BIN5518
Stictopisthus sp.|EE-7-89 MP|Dolichogenidea absona|
Stictopisthus sp.|EE-18-94 MP|Dolichogenidea absona|
Ichneumonidae|EE-10169-86 P2|Epinota radicana|
Exochus lictor docoratus|EE-700-93 P3|Acleris variana|BIN10400
Exochus lictor docoratus|EE-6074-88 P2|Acleris variana|BIN10400
Apechthis ontario|EE-6213-88 P2|Acleris variana|BIN10400
Exochus lictor docoratus|EE-13599-86 P2|Acleris variana|BIN10400
Exochus lictor decoratus|EE-13234-86R P2|Acleris variana|BIN10400
Exochus lictor decoratus|EE-6851-88R P2|Acleris variana|BIN10400
Exochus lictor decoratus|EE-7726-87R P2|Acleris variana|BIN10400
Exochus lictor decoratus|EE-6789-88R P2|Coleotechnites piceaella|BIN10400
Scambus sp.|EE-4146-90 P3|Acleris variana|BIN10400
Itoplectis conquisitor|EE-5072-88 P2|Coleotechnites piceaella|BIN11282
Itoplectis conquisitor|EE-5719-88R P2|Coleotechnites piceaella|BIN11282
Itoplectis conquisitor|EE-6943-88R P2|Acleris variana|BIN11282
Itoplectis conquisitor|EE-5202-88 P2|Coleotechnites piceaella|BIN11283
Itoplectis conquisitor|EE-6859-88R P2|Coleotechnites piceaella|BIN11283
Itoplectis conquisitor|EE-13428-86 P2|Choristoneura fumiferana|BIN11281
Itoplectis conquisitor|EE-17623-85 P1|Choristoneura fumiferana|BIN11281
Itoplectis sp.|EE-607-85 P1|Glypta fumiferanae|BIN11281
Itoplectis sp.|EE-1359-85 P1|Glypta fumiferanae|BIN11281
Itoplectis conquisitor|EE-17546-85 P1|Choristoneura fumiferana|BIN11281
Itoplectis conquisitor|EE-5897-88 P3|Choristoneura fumiferana|BIN11281
Itoplectis conquisitor|EE-6230-88 P3|Choristoneura fumiferana|BIN11281
Itoplectis conquisitor|EE-5658-88 P3|Choristoneura fumiferana|BIN11281
Itoplectis conquisitor|EE-6679-88 P3|Acleris variana|BIN11281
Itoplectis conquisitor|EE-6703-88 P3|Choristoneura fumiferana|BIN11281
Itoplectis sp.|EE-520-88 P3|Glypta fumiferanae|BIN11281
Itoplectis conquisitor|EE-18215-85 P1|Choristoneura fumiferana|BIN11281

[illegible]
Itoplectis conquisitor|EE-6703-88 P3|Choristoneura fumiferana|BIN11281
Itoplectis sp.|EE-520-88 P3|Glypta fumiferanae|BIN11281
Itoplectis conquisitor|EE-18215-85 P1|Choristoneura fumiferana|BIN11281
Itoplectis conquisitor|EE-18600-85 P1|Choristoneura fumiferana|BIN11281
Itoplectis conquisitor|EE-17201-85 P1|Choristoneura fumiferana|BIN11281
Itoplectis conquisitor|EE-6560-88 P3|Choristoneura fumiferana|BIN11281
Itoplectis conquisitor|EE-17464-85 P1|Choristoneura fumiferana|BIN11281
Itoplectis conquisitor|EE-17803-85 P1|Choristoneura fumiferana|BIN11281
Itoplectis sp.|EE-235-90 P3|Glypta fumiferanae|BIN11281
Itoplectis conquisitor|EE-6348-88 P3|Choristoneura fumiferana|BIN11281
Itoplectis conquisitor|EE-6170-88 P3|Choristoneura fumiferana|BIN11281
Itoplectis conquisitor|EE-6755-88 P3|Choristoneura fumiferana|BIN11281
Itoplectis conquisitor|EE-6610-88 P3|Choristoneura fumiferana|BIN11281
Itoplectis conquisitor|EE-12856-86 P2|Choristoneura fumiferana|BIN11281
Itoplectis conquisitor|EE-17881-85 P1|Choristoneura fumiferana|BIN11281
Itoplectis conquisitor|EE-16602-85 P1|Choristoneura fumiferana|BIN11281
Itoplectis conquisitor|EE-6128-88 P3|Choristoneura fumiferana|BIN11281
Itoplectis sp.|EE-361-85 P1|Glypta fumiferanae|BIN11281
Itoplectis conquisitor|EE-16611-85 P1|Choristoneura fumiferana|BIN11281
Itoplectis sp.|EE-1800-85 P1|Glypta fumiferanae|
Apechthis ontario|EE-9995-86 P2|Choristoneura fumiferana|BIN9338
Apechthis ontario|EE-17378-85 P1|Choristoneura fumiferana|BIN9338
Apechthis ontario|EE-12333-86 P2|Choristoneura fumiferana|BIN9338
Apechthis ontario|EE-12646-86 P2|Choristoneura fumiferana|BIN9338
Apechthis ontario|EE-13189-86 P2|Choristoneura fumiferana|BIN9338
Apechthis ontario|EE-6178-91 P3|Acleris variana|BIN9338
Apechthis ontario|EE-16238-85 P1|Choristoneura fumiferana|BIN9338
Apechthis ontario|EE-17552-85 P1|Choristoneura fumiferana|BIN9338
Apechthis ontario|EE-6561-88 P3|Choristoneura fumiferana|BIN9338
Apechthis ontario|EE-19948-85 P1|Choristoneura fumiferana|BIN9338
Apechthis ontario|EE-10755-86 P2|Choristoneura fumiferana|BIN9338
Apechthis ontario|EE-11521-86 P2|Choristoneura fumiferana|BIN9338
Apechthis ontario|EE-5653-88 P3|Choristoneura fumiferana|BIN9338
Apechthis ontario|EE-6526-88 P3|Choristoneura fumiferana|BIN9338
Apechthis ontario|EE-6358-88 P3|Choristoneura fumiferana|BIN9338
Apechthis ontario|EE-6104-88 P3|Choristoneura fumiferana|BIN9338
Apechthis ontario|EE-5803-88 P3|Choristoneura fumiferana|BIN9338
Apechthis ontario|EE-13296-86 P2|Choristoneura fumiferana|BIN9338
Apechthis ontario|EE-13250-86 P2|Choristoneura fumiferana|BIN9338
Apechthis ontario|EE-9940-86 P2|Choristoneura fumiferana|BIN9338
Apechthis ontario|EE-16960-85 P1|Choristoneura fumiferana|BIN9338
Apechthis ontario|EE-15958-85 P1|Choristoneura fumiferana|BIN9338
Apechthis ontario|EE-17340-85 P1|Choristoneura fumiferana|BIN9338
Apechthis ontario|EE-16313-85 P1|Choristoneura fumiferana|BIN9338
Apechthis ontario|EE-17419-85 P1|Choristoneura fumiferana|BIN9338
Apechthis ontario|EE-10842-86 P2|Choristoneura fumiferana|BIN9338
Apechthis ontario|EE-6485-88 P3|Choristoneura fumiferana|BIN9338
Apechthis ontario|EE-6590-88 P3|Choristoneura fumiferana|BIN9338
Apechthis ontario|EE-6638-88 P3|Choristoneura fumiferana|BIN9338
Apechthis ontario|EE-6918-88 P3|Choristoneura fumiferana|BIN9338
Dirophanes hariolus|EE-6910-88 P3|Choristoneura fumiferana|BIN9338
Pimpla pedalis|EE-3783-90 P3|Choristoneura fumiferana|BIN35192
Pimpla sp.|EE-3520-90 P3|Choristoneura fumiferana|BIN35192
Scambus sp.|EE-1021-85 P1|Glypta fumiferanae|BIN10893
Scambus sp.|EE-1034-85 P1|Glypta fumiferanae|BIN10893
Scambus sp.|EE-568-88 P3|Glypta fumiferanae|BIN10893
Scambus sp.|EE-1676-85 P1|Glypta fumiferanae|BIN10893
Scambus sp.|EE-356-85 P1|Glypta fumiferanae|BIN10893
Scambus sp.|EE-567-85 P1|Glypta fumiferanae|BIN10893
Scambus sp.|EE-1365-85 P1|Glypta fumiferanae|BIN10893
Scambus sp.|EE-1382-85 P1|Glypta fumiferanae|BIN10893
Scambus sp.|EE-1123-86 P2|Meteorus trachynotus|BIN10893
Scambus hispae|EE-4310-90R P3|Choristoneura fumiferana|BIN10892
Scambus sp.|EE-4292-90R P3|Choristoneura fumiferana|BIN10892
Scambus hispae|EE-4445-90R P3|Choristoneura fumiferana|BIN10892
Scambus sp.|EE-6656-88 P3|Choristoneura fumiferana|
Scambus sp.|EE-6915-88 P3|Choristoneura fumiferana|BIN10892
Scambus sp.|EE-3794-92 P3|Choristoneura fumiferana|BIN10892
Scambus sp.|EE-H190-88 P3|Apanteles sp.|
Scambus sp.|EE-102-86 P2|Glypta fumiferanae|BIN10892
Scambus sp.|EE-H283-91 P3|Apanteles fumiferanae|BIN10892
Scambus sp.|EE-807-86 P2|Meteorus trachynotus|BIN10892
Scambus hispae|EE-E39-95 P2|Choristoneura fumiferana|BIN49484
Scambus hispae|EE-C22-95 P2|Choristoneura fumiferana|BIN49484
Scambus sp.|EE-1313-85 P1|Glypta fumiferanae|BIN10894
Scambus sp.|EE-1497-89 P3|Glypta fumiferanae|BIN10894
Scambus sp.|EE-1453-89 P3|Glypta fumiferanae|BIN10894
Scambus sp.|EE-1533-89 P3|Glypta fumiferanae|BIN10894
Scambus sp.|EE-379-90 P3|Glypta fumiferanae|BIN10894
Scambus sp.|EE-426-90 P3|Glypta fumiferanae|BIN10894
Scambus sp.|EE-468-90 P3|Glypta fumiferanae|BIN10894
Scambus sp.|EE-1086-85 P1|Glypta fumiferanae|BIN10894
Scambus sp.|EE-1321-85 P1|Glypta fumiferanae|BIN10894
Scambus sp.|EE-209-87 P2|Glypta fumiferanae|BIN10894
Scambus sp.|EE-401-87 P2|Glypta fumiferanae|BIN10894
Scambus sp.|EE-929-88 P3|Glypta fumiferanae|BIN10894
Scambus decorus|EE-H489-84 P1|Glypta fumiferanae|BIN10894
Scambus sp.|EE-673-89 P3|Glypta fumiferanae|BIN10894
Scambus sp.|EE-1476-88 P3|Glypta fumiferanae|BIN10894
Ischnus inquisitorius|EE-E6-95 P2|Choristoneura fumiferana|BIN67737
Acrolyta sp.|EE-147-92 P3|Apanteles fumiferanae|BIN48196
Acrolyta sp.|EE-630-88 P3|Apanteles fumiferanae|BIN48196
Acrolyta sp.|EE-557-90 P3|Glyptapanteles sp.|BIN6282
Acrolyta sp.|EE-H177-89 P2|Glyptapanteles sp.|BIN6282
Gelis sp.|EE-185-87 P2|Apanteles fumiferanae|BIN6282
Gelis sp.|EE-H384-90 P3|Apanteles fumiferanae|BIN6277
Gelis sp.|EE-186-88 P3|Apanteles fumiferanae|
Gelis sp.|EE-423-85 P1|Apanteles fumiferanae|BIN6280
Gelis sp.|EE-1238-84 P1|Meteorus trachynotus|BIN6280
Gelis sp.|EE-29-90 MP|Dolichogenidea absona|BIN6280
Gelis sp.|EE-954-88 P3|Apanteles fumiferanae|BIN6280
Gelis sp.|EE-414-88 P3|Apanteles fumiferanae|BIN6280

Gelis sp.|EE-29-90 MP|Dolichogenidea absona|BIN6280
Gelis sp.|EE-954-88 P3|Apanteles fumiferanae|BIN6280
Gelis sp.|EE-414-88 P3|Apanteles fumiferanae|BIN6280
Gelis sp.|EE-48-87 P2|Apanteles fumiferanae|BIN6280
Gelis sp.|EE-21-88 MP|Apanteles petrovae|BIN6280
Gelis sp.|EE-228-91 P3|Glypta fumiferanae|BIN6280
Gelis sp.|EE-1051-88 P3|Apanteles fumiferanae|BIN6280
Gelis sp.|EE-635-88 P3|Apanteles fumiferanae|BIN6280
Gelis sp.|EE-424-88 P3|Apanteles fumiferanae|BIN6280
Gelis sp.|EE-417-88 P3|Apanteles fumiferanae|BIN6280
Gelis sp.|EE-388-88 P3|Apanteles fumiferanae|BIN6280
Gelis sp.|EE-95-88 P3|Apanteles fumiferanae|BIN6280
Gelis sp.|EE-68-87 P2|Apanteles fumiferanae|BIN6280
Gelis sp.|EE-368-92 P3|Glytapanteles sp.|BIN6280
Gelis sp.|EE-1452-89 P3|Meteorus trachynotus|BIN6280
Gelis sp.|EE-1169-89 P3|Meteorus trachynotus|BIN6280
Gelis sp.|EE-62-86 P2|Apanteles fumiferanae|BIN6280
Gelis sp.|EE-59-86 P2|Apanteles fumiferanae|BIN6280
Gelis sp.|EE-18-90 MP|Dolichogenidea absona|BIN6280
Gelis sp.|EE-128-88 MP|Apanteles morrisi|BIN6280
Gelis sp.|EE-489-86 P2|Apanteles fumiferanae|BIN6280
Gelis sp.|EE-267-85 P1|Apanteles fumiferanae|BIN6280
Gelis sp.|EE-12-90 MP|Dolichogenidea absona|BIN6280
Gelis sp.|EE-30-90 MP|Dolichogenidea absona|BIN6280
Gelis sp.|EE-14-91 MP|Apanteles petrovae|BIN6280
Gelis sp.|EE-494-86 P2.2|Apanteles fumiferanae|BIN6280
Gelis sp.|EE-150-89 MP|Apanteles morrisi|BIN6280
Gelis sp.|EE-50-89 MP|Apanteles morrisi|BIN6280
Gelis sp.|EE-34-90 MP|Dolichogenidea absona|BIN6280
Gelis sp.|EE-52-87 P2|Apanteles fumiferanae|BIN6281
Gelis sp.|EE-H117-87 P1|Apanteles sp.|
Gelis sp.|EE-H157-87 P1|Apanteles sp.|
Gelis sp.|EE-854-92 P3|Glytapanteles sp.|BIN6283
Gelis sp.|EE-1239-89 P3|Meteorus trachynotus|BIN6283
Gelis sp.|EE-21-93 P3|Apanteles sp.|BIN6283
Gelis sp.|EE-5-93 P3|Apanteles sp.|BIN6283
Gelis sp.|EE-992-92 P3|Glytapanteles sp.|BIN6283
Gelis sp.|EE-H9-91 P3|Apanteles sp.|
Gelis sp.|EE-51-93 MP|Apanteles petrovae|BIN6278
Gelis sp.|EE-109-85 P1|Apanteles fumiferanae|BIN6278
Gelis sp.|EE-122-85 P1|Apanteles fumiferanae|BIN6278
Gelis sp.|EE-385-85 P1|Apanteles fumiferanae|BIN6278
Gelis sp.|EE-H413-91 P3|Apanteles sp.|BIN6278
Gelis sp.|EE-120-95 MP|Apanteles sp.|BIN6278
Gelis sp.|EE-31-89 MP|Dolichogenidea absona|BIN6279
Gelis sp.|EE-11-88 MP|Apanteles morrisi|BIN6279
Gelis sp.|EE-86-85 P1|Apanteles fumiferanae|BIN6279
Gelis sp.|EE-1088-89 P3|Meteorus trachynotus|BIN6279
Gelis sp.|EE-34-85 P1|Apanteles fumiferanae|BIN6279
Gelis sp.|EE-614-85 P1|Apanteles fumiferanae|BIN6279
Gelis sp.|EE-4-90 MP|Dolichogenidea absona|BIN6279
Gelis sp.|EE-H758-91 P3|Apanteles sp.|BIN6279
Gelis sp.|EE-63-93 MP|Apanteles petrovae|BIN6279
Gelis sp.|EE-32-93 P3|Apanteles sp.|BIN6279
Coelichneumon sp.|EE-29-84R P2|Choristoneura fumiferana|BIN67679
Dirophanes hariolus|EE-13275-88 P2|Coleotechnites piceaella|BIN9337
Dirophanes hariolus|EE-17981-85 P1|Choristoneura fumiferana|BIN9337
Dirophanes hariolus|EE-9937-86 P2|Choristoneura fumiferana|BIN9337
Dirophanes hariolus|EE-6381-88 P3|Choristoneura fumiferana|BIN9337
Dirophanes sp.|EE-13651-86 P1|Choristoneura fumiferana|BIN9337
Dirophanes sp.|EE-13197-86 P2|Choristoneura fumiferana|BIN9337
Dirophanes sp.|EE-12768-86 P2|Choristoneura fumiferana|BIN9337
Dirophanes sp.|EE-12499-86 P2|Choristoneura fumiferana|BIN9337
Dirophanes sp.|EE-13352-86 P1|Choristoneura fumiferana|BIN9337
Dirophanes sp.|EE-12119-86 P1|Choristoneura fumiferana|BIN9337
Dirophanes sp.|EE-11850-86 P1|Choristoneura fumiferana|BIN9337
Dirophanes sp.|EE-18969-85 P1|Choristoneura fumiferana|BIN9337
Dirophanes sp.|EE-18930-85 P1|Choristoneura fumiferana|BIN9337
Dirophanes sp.|EE-18837-85 P1|Choristoneura fumiferana|BIN9337
Dirophanes hariolus|EE-6741-88 P3|Choristoneura fumiferana|BIN9337
Dirophanes hariolus|EE-6650-88 P3|Choristoneura fumiferana|BIN9337
Dirophanes hariolus|EE-6635-88 P3|Choristoneura fumiferana|BIN9337
Dirophanes hariolus|EE-6449-88 P3|Choristoneura fumiferana|BIN9337
Dirophanes hariolus|EE-6444-88 P3|Choristoneura fumiferana|BIN9337
Dirophanes hariolus|EE-6107-88 P3|Choristoneura fumiferana|BIN9337
Dirophanes hariolus|EE-13593-86 P2|Choristoneura fumiferana|BIN9337
Dirophanes hariolus|EE-13080-86 P2|Choristoneura fumiferana|BIN9337
Dirophanes hariolus|EE-11122-86 P2|Choristoneura fumiferana|BIN9337
Dirophanes hariolus|EE-18891-85 P1|Choristoneura fumiferana|BIN9337
Dirophanes hariolus|EE-18756-85 P1|Choristoneura fumiferana|BIN9337
Dirophanes hariolus|EE-16261-85 P1|Choristoneura fumiferana|BIN9337
Dirophanes hariolus|EE-6268-88 P3|Choristoneura fumiferana|BIN9337
Dirophanes hariolus|EE-13597-86 P2|Choristoneura fumiferana|BIN9337
Dirophanes hariolus|EE-12834-86 P2|Choristoneura fumiferana|BIN9337
Dirophanes hariolus|EE-19016-85 P1|Choristoneura fumiferana|BIN9337
Dirophanes hariolus|EE-18915-85 P1|Choristoneura fumiferana|BIN9337
Dirophanes hariolus|EE-18724-85 P1|Choristoneura fumiferana|BIN9337
Dirophanes hariolus|EE-18720-85 P1|Choristoneura fumiferana|BIN9337
Dirophanes hariolus|EE-11513-86 P2|Choristoneura fumiferana|BIN9337
Dirophanes hariolus|EE-6466-88 P3|Choristoneura fumiferana|BIN9337
Dirophanes hariolus|EE-13274-88 P2|Coleotechnites piceaella|BIN9337
Dirophanes hariolus|EE-13278-88 P2|Coleotechnites piceaella|BIN9337
Dirophanes sp.|EE-12812-86 P2|Choristoneura fumiferana|BIN9337
Dirophanes hariolus|EE-12602-86 P2|Choristoneura fumiferana|BIN9337
Dirophanes hariolus|EE-13302-86 P2|Choristoneura fumiferana|BIN9337
Dirophanes hariolus|EE-18916-85 P1|Choristoneura fumiferana|BIN9337
Dirophanes hariolus|EE-18815-85 P1|Choristoneura fumiferana|BIN9337
Dirophanes hariolus|EE-11333-86 P2|Choristoneura fumiferana|BIN9337
Apechthis ontario|EE-18043-85 P1|Choristoneura fumiferana|BIN9337
Dirophanes sp.|EE-12546-86 P1|Choristoneura fumiferana|BIN9337
Dirophanes sp.|EE-13240-86 P2|Choristoneura fumiferana|BIN9337
Dirophanes hariolus|EE-6055-91 P3|Coleotechnites piceaella|BIN9337
Apechthis ontario|EE-6681-88 P2|Acleris variana|BIN9337

Dirophanes sp.|EE-[illegible]|Choristoneura fumiferana|BIN9337
Dirophanes sp.|EE-13240-86 P2|Choristoneura fumiferana|BIN9337
Dirophanes hariolus|EE-6055-91 P3|Coleotechnites piceaella|BIN9337
Apechthis ontario|EE-6681-88 P2|Acleris variana|BIN9337
Dirophanes sp.|EE-6699-88R P3|Acleris variana|BIN9337
Phaeogenes sp.|EE-4965-89 P2|Acleris variana|BIN9337
Dirophanes sp.|EE-6938-88R P2|Acleris variana|
Itoplectis conquisitor|EE-3888-92 P3|Acleris variana|BIN9337
Dirophanes hariolus|EE-4613-89 P2|Acleris variana|BIN9337
Dirophanes hariolus|EE-6142-91 P3|Acleris variana|BIN9337
Dirophanes hariolus|EE-3889-92 P3|Acleris variana|BIN9337
Dirophanes sp.|EE-7001-88R P2|Acleris variana|BIN9337
Dirophanes sp.|EE-6463-88R P3|Acleris variana|BIN9337
Phaeogenes sp.|EE-13085-86 P2|Acleris variana|BIN9337
Phaeogenes sp.|EE-4951-89 P3|Acleris variana|BIN9337
Gelis sp.|EE-H859-91 P3|Glypta sp.|BIN6276
Mastrus sp.|EE-396-89 P3|Glypta fumiferanae|BIN6276
Gelis sp.|EE-H55-87 P1|Glypta fumiferanae|BIN6276
Gelis sp.|EE-H181-87 P1|Glypta fumiferanae|BIN6276
Mastrus sp.|EE-1574-84 P1|Glypta fumiferanae|BIN6276
Mastrus sp.|EE-1198-91 P3|Scambus sp. Via Glypta fumiferanae|BIN6276
Mastrus sp.|EE-345-92 P3|Glypta fumiferanae|BIN6276
Mastrus sp.|EE-61-87 P1|Glypta fumiferanae|BIN6276
Mastrus sp.|EE-795-86 P1|Glypta fumiferanae|BIN6276
Campoplex sp.|EE-7179-87 P2|Coleotechnites piceaella|BIN47917
Campoplex sp.|EE-3821-92 P3|Epinota radicana|BIN47918
Campoplex sp.|EE-5394-91R P3|Epinota radicana|BIN47918
Campoplex sp.|EE-P173-90 P2|Choristoneura fumiferana|BIN47919
Campoplex sp.|EE-286-94 MP|Choristoneura fumiferana|
Campoplex sp.|EE-1062-93 MP|Choristoneura fumiferana|
Enytus montanus|EE-5555-87 P2|Choristoneura fumiferana|BIN31926
Tranosema rostrale|EE-9-83R P2|Choristoneura fumiferana|BIN31926
Tranosema rostrale|EE-3374-90R P2|Choristoneura fumiferana|BIN31926
Tranosema rostrale|EE-5079-87 P2|Choristoneura fumiferana|BIN31926
Tranosema rostrale|EE-5168-87 P1|Choristoneura fumiferana|BIN31926
Tranosema rostrale|EE-2554-92 P3|Choristoneura fumiferana|BIN31926
Tranosema rostrale|EE-2557-92 P3|Choristoneura fumiferana|BIN31926
Diadegma stenostomus group|EE-492-92 P2|Coleotechnites piceaella|BIN18765
Diadegma sp.|EE-2951-89 P2|Coleotechnites piceaella|BIN18765
Diadegma sp.|EE-1192-92 P2|Coleotechnites piceaella|BIN18765
Diadegma sp.|EE-5378-88 P1|Coleotechnites piceaella|BIN18765
Diadegma sp.|EE-3121-89 P1|Coleotechnites piceaella|BIN18765
Diadegma sp.|EE-4439-88 P2|Coleotechnites piceaella|BIN18765
Diadegma sp.|EE-4935-88 P2|Coleotechnites piceaella|BIN18765
Diadegma sp.|EE-2077-92 P2|Coleotechnites piceaella|BIN18765
Diadegma sp.|EE-4692-88 P1|Coleotechnites piceaella|BIN18765
Diadegma sp.|EE-330-88 P2|Coleotechnites piceaella|BIN18765
Diadegma sp.|EE-3827-89 P2|Coleotechnites piceaella|BIN18765
Diadegma sp.|EE-3411-91 P2|Coleotechnites piceaella|BIN18765
Diadegma sp.|EE-2185-89 P3|Choristoneura fumiferana|BIN18765
Diadegma sp.|EE-3987-90 P3|Coleotechnites piceaella|BIN18765
Diadegma sp.|EE-4322-90 P3|Coleotechnites piceaella|BIN18765
Diadegma stenostomus group|EE-637-93 P3|LEPIDOPTERA|BIN18765
Enytus montanus|EE-H226-91 P3|Choristoneura fumiferana|BIN18766
Enytus montanus|EE-H80-88 P3|Choristoneura fumiferana|BIN18766
Enytus montanus|EE-H441-88 P3|Choristoneura fumiferana|BIN18766
Enytus montanus|EE-4677-88 P1|Choristoneura fumiferana|BIN18766
Diadegma sp.|EE-3526-89 P3|Choristoneura fumiferana|BIN18766
Enytus montanus|EE-4238-88 P3|Choristoneura fumiferana|BIN18766
Diadegma sp.|EE-2990-89 P3|Choristoneura fumiferana|BIN18766
Enytus montanus|EE-4916-88 P2|Choristoneura fumiferana|
Enytus montanus|EE-5849-88 P3|Choristoneura fumiferana|BIN18766
Hyposoter sp.|EE-H258-91 P3|unknown|BIN46046
Tranosema tenuifemur|EE-660-93 MP|Choristoneura fumiferana|BIN13351
Tranosema tenuifemur|EE-4574-88 P3|Choristoneura fumiferana|BIN13351
Tranosema tenuifemur|EE-3304-87 P1|Choristoneura fumiferana|BIN13351
Tranosema tenuifemur|EE-3028-89R P3|Choristoneura fumiferana|BIN13351
Tranosema tenuifemur|EE-5364-88 P3|Choristoneura fumiferana|BIN13351
Tranosema tenuifemur|EE-135-91 MP|Choristoneura fumiferana|BIN13351
Tranosema tenuifemur|EE-725-93 MP|Choristoneura fumiferana|BIN13351
Tranosema tenuifemur|EE-822-93 MP|Choristoneura fumiferana|BIN13351
Tranosema tenuifemur|EE-2272-89R P2|Choristoneura fumiferana|BIN13351
Tranosema tenuifemur|EE-1101PL-93-P2|Choristoneura fumiferana|BIN13351
Tranosema tenuifemur|EE-111-90 MP|Choristoneura fumiferana|BIN13351
Tranosema tenuifemur|EE-323-93 MP|Choristoneura fumiferana|BIN13351
Tranosema tenuifemur|EE-3-93 MP|Choristoneura fumiferana|BIN13351
Tranosema tenuifemur|EE-2826-89R P2|Choristoneura fumiferana|BIN13351
Tranosema tenuifemur|EE-P603-89 P2|Choristoneura fumiferana|BIN13351
Tranosema tenuifemur|EE-4618-88 P1|Choristoneura fumiferana|BIN13351
Tranosema tenuifemur|EE-2838-89R P2|Choristoneura fumiferana|BIN13351
Tranosema tenuifemur|EE-3925-89R P3|Choristoneura fumiferana|BIN13351
Tranosema tenuifemur|EE-758PL-93 P2|Choristoneura fumiferana|BIN13351
Tranosema tenuifemur|EE-688-93 P3|Acleris variana|BIN13351
Tranosema tenuifemur|EE-532-93 P3|Choristoneura fumiferana|BIN13351
Tranosema tenuifemur|EE-5839-88 P3|Choristoneura fumiferana|BIN13351
Tranosema tenuifemur|EE-2558-89R P3|Choristoneura fumiferana|BIN13351
Tranosema tenuifemur|EE-301-93 MP|Choristoneura fumiferana|
Tranosema tenuifemur|EE-580-93 MP|Choristoneura fumiferana|BIN13351
Tranosema tenuifemur|EE-855-93 MP|Choristoneura fumiferana|BIN13351
Trathala recurvariae|EE-4968-88 P2|Coleotechnites piceaella|
Pristomerus sp.|EE-1385-86 P1|Choristoneura fumiferana|
Trathala recurvariae|EE-2925-90R P3|Coleotechnites piceaella|
Trathala recurvariae|EE-3151-89R P1|Coleotechnites piceaella|
Trathala recurvariae|EE-2049-92 P3|Coleotechnites piceaella|BIN98584
Trathala recurvariae|EE-5702-88 P2|Acleris variana|
Trathala recurvariae|EE-4163-89R P2|Acleris variana|
Trathala recurvariae|EE-5917-88 P1|Acleris variana|
Pristomerus baumhoferi|EE-P220-89 P2|Choristoneura fumiferana|
Pristomerus baumhoferi|EE-P554-89 P2|Choristoneura fumiferana|
Trathala recurvariae|EE-310-94 MP|Acleris variana|
Trathala recurvariae|EE-236-95 MP|Coleotechnites piceaella|
