# Supplementary material for: Barcoding a Quantified Food Web: Crypsis, Concepts, Ecology and Hypotheses
Source: PLoS One. 2011 Jul 6;6(7):e14424. doi: 10.1371/journal.pone.0014424 (PMC3130735; doi:10.1371/journal.pone.0014424)
Supplement: Table S4 — VanVeen ITS2 alignment/structure and CBC table from GenBank specimens: AJ309962-AJ309965. (DOC) [file pone.0014424.s005.doc]

**SI Table 4: VanVeen ITS2 alignment/structure and CBC table from GenBank specimens: AJ309962-AJ309965.**

>AJ309962| Alloxysta victrix internal transcribed spacer 2, ITS2

GGGTCGTTTATAAATTAAAGACTGCTTATCTCTCTTATAGAGAGATACGAGCGAAAAATGAACGTTTGTCACAATATTTTGTTTTTCATTCATTTGAAATAATCAAAGTTGTGGCGTCGTTTGAAATAAGTTGAGAAAAAACGTTCCTTGACACACAAAACATAGCCGTCCAGGTCTGTCTAGTACAGATTTTGAACGCAATCACGTGCACAAATTCATGTGTTTTGATAGTGAATTGGTTGTGTCGAGTCCCGGAGTTAAAATATTTTATTTTGCACGGATCGATATCAAATCATTTTCATGTTTCATTACATATTGTGTACAAATGTGATTTTAAAAATTTTTGGACGGTTTGTGTTTAATTTCAAGTGTAACTTAATATTTTTTTCTT----------------

.((((.((((.....))))))))(((((((((((.....)))))))..)))).......(((((..(((((((((..((((..(((((......)))))....))))))))))))).)))))..........((((((((..(((((((((......(((((((((((((((.((((((.....)))))).........(((((((((((((.....((((((..(((..(((((.((((((((((((...(((..((.((((((...)))))))).))).)))))))..)))))..)))))...)))..)))))))))))))....))))))...........))))))).))))))))....))))).).)))......))))))))..----------------

>AJ309963| Alloxysta leunisii internal transcribed spacer 2, ITS2

GGGTCGTTTATAAATTAAAGACTGCTTATCTTGTTATAAAGATACGAGCGAAAAATGAACGTTCGTCACAGTTTTGTTTTTCATTTATTTGAAATATAATAAAAGTTGTGGCGTCGTTTGAAATAAGTTGAGAAAAAAACGTTCCTTGACACACAAAACATAGCCGTCCAGGTCTGTCTAGTACAGATTTTGAACGCAATCACGTGCACAAATTCATGTGTTTTGATAGTGAATTGGTTGTGTCGAGTCCCGGAGTTAAAATATTTTATTTTGCACGGATCGATATCAAATCATTTTCATGTTTCATTACATATTGTGTACAAATGTGATTTTTTAAAATTTTTGGACGGTTTGTGTATGTGTTTGTTTCAAGTGTAACTTAATATTTTTTTCTT------------

.((((.((((.....))))))))(((((((((......)))))..)))).......(((((..((((((((((((((((((((......)))))...))))))).)))))))).)))))..........((((((((((.(((((((((.(((((..(((((((((((((.((((((.....))))))((((((...(((((((((((((.....((((((..(((..(((((.((((((((((((...(((..((.((((((...)))))))).))).)))))))..)))))..)))))...)))..)))))))))))))....)))))).)))))).....))))))).)))))).))))).....))))).).)))......))))))))))------------

>AJ309964| Alloxysta fuscicornis internal transcribed spacer 2, ITS2

GGGTCGTTTATAAATTAAAGACTGCTTATCTCTCTTATAAAGAGAAGATACGAGCGAAAAATGAACGTTCGTCACAATATTTTGTTTTTCATTTATTTGAAATAATCAAAGTTGTGGCGTCGTTTGAAATAAGTTGAGAAAAAAACGTTCCTTGACACACACAAAACATAGCCGTCCAGGTCTGTCTAGTACAGATTTTGAACGCAATCACGTGCACAAATTCATGTGTTTTGATAGTGAATTGGTTGTGTCGAGTCCCGGAGTTAAAATATTTTATTTTGCACGGATCGATATCAAATCATTTTCATGTTTCATTACATATTGTGTACAAATGTGATTTTTTAAAATTTTTGGACGGTTTGTGTATGTGTGATGGTTTCAAGTGTAACTTAATATATTTTTCTT--

.((((.((((.....))))))))((((((((((((.....)))).))))..))))((((((((.(((..(((((((((..((((..(((((......)))))....))))))))))))).)))(((((((..((..((............))..)).((((((..(((((((((((((.((((((.....))))))((((((...(((((((((((((.....((((((..(((..(((((.((((((((((((...(((..((.((((((...)))))))).))).)))))))..)))))..)))))...)))..)))))))))))))....)))))).)))))).....))))))).)))))).))))))...))))))).............))))))))..--

>AJ309965| Alloxysta tscheki internal transcribed spacer 2, ITS2

GGGTCGTTTATAAATTAAAGACTGCTTATCTTCTCTCTTATAGAGAGAAGATACGAGCGAAAAATGAACGTTCGTCACAATATTTTGTTTTTCATTTATTTGAAATAATCAAAGTTGTGGCGTCGTTTGAAATAAGTTGAGAAAAAAACGTTCCTTGACACACACAAAACATAGCCGTCCAGGTCTGTCTAGTACAGATTTTGAACGCAATCACGTGCACAAATTTATGTGTTTTGATAGTGAATTGGTTGTGTCGAGTCCCGGAGTTAAAATATTTTATTTTGCACGGATCGATATCAAATCATTTTCATGTTTCATTACATATTGTGTACAAATGTGATTTTTAAAAATTTTTGGACGGTTTGTGTTTGTGTTTAATTTCAAGTGTAACTTAATATTTTTTTCTT

.((((.((((.....))))))))((((((((((((((.....))))))))))..)))).......(((((..(((((((((..((((..(((((......)))))....))))))))))))).)))))..........((((((((((..((.....(((((((((((.(((((((((((((.(((((.....)))))(((((((...(((((((((((((.....((((((..(((..(((((.((((((((((((...(((..((.((((((...)))))))).))).)))))))..)))))..)))))...)))..)))))))))))))....)))))))))))))....)))))))).))))))))))))..........)))).....))..))))))))))

**CBC matrix**
